# The centrosomal OFD1 protein interacts with the translation machinery and regulates the synthesis of specific targets

**DOI:** 10.1038/s41598-017-01156-x

**Published:** 2017-04-27

**Authors:** Daniela Iaconis, Maria Monti, Mario Renda, Arianne van Koppen, Roberta Tammaro, Marco Chiaravalli, Flora Cozzolino, Paola Pignata, Claudia Crina, Piero Pucci, Alessandra Boletta, Vincenzo Belcastro, Rachel H. Giles, Enrico Maria Surace, Simone Gallo, Mario Pende, Brunella Franco

**Affiliations:** 1 Telethon Institute of Genetics and Medicine (TIGEM), Via Campi Flegrei, 34, 80078 Pozzuoli, Naples Italy; 2 Dipartimento di Scienze Chimiche and CEINGE Biotecnologie Avanzate, Università di Napoli Federico II, Via Gaetano Salvatore 482, 80145 Napoli, Italy; 3 Department of Nephrology and Hypertension, University Medical Center Utrecht, Heidelberglaan 100, 3584CX Utrecht, The Netherlands; 4 Division of Genetics and Cell Biology, Dibit, San Raffaele Scientific Institute, Via Olgettina, 58 – 20132 Milan, Italy; 5 Molecular Histology and Cell Growth Unit, INGM - Istituto Nazionale di Genetica Molecolare “Romeo and Enrica Invernizzi”, Via Francesco Sforza, 35 – 20122, Milan, Italy; 6 Institut National de la Santé et de la Recherche Médicale (INSERM) U1151, Institut Necker Enfants Malades, Université Paris Descartes, Sorbonne Paris Cité, Paris, France; 7 Department of Translational Medicine, University of Naples “Federico II”, Via Sergio Pansini, 80131 Naples, Italy

## Abstract

Protein synthesis is traditionally associated with specific cytoplasmic compartments. We now show that OFD1, a centrosomal/basal body protein, interacts with components of the Preinitiation complex of translation (PIC) and of the eukaryotic Initiation Factor (eIF)4F complex and modulates the translation of specific mRNA targets in the kidney. We demonstrate that OFD1 cooperates with the mRNA binding protein Bicc1 to functionally control the protein synthesis machinery at the centrosome where also the PIC and eIF4F components were shown to localize in mammalian cells. Interestingly, Ofd1 and Bicc1 are both involved in renal cystogenesis and selected targets were shown to accumulate in two models of inherited renal cystic disease. Our results suggest a possible role for the centrosome as a specialized station to modulate translation for specific functions of the nearby ciliary structures and may provide functional clues for the understanding of renal cystic disease.

## Introduction

The initiation of mRNA translation in eukaryotes is an articulated process composed of different steps. Among these, the formation of the Preinitiation complex (PIC) and of the eIF4F complex is finely regulated. Specific translation factors contribute to the modulation of initiation by interacting with the ribosome, the mRNA and/or other translation factors. In particular, the PIC includes the 40S ribosomal subunit and several initiation factors, like eIF2 and eIF3; then, eIF4E, eIF4A and eIF4G associates with the PIC to form the eIF4F complex and promote Cap-dependent translation^1–3^. Signaling pathways can impact translation at multiple steps. For instance, the phosphorylation status of eIF2α and eIF4E availability is rate limiting for translation efficiency^4, 5^. mTORC1 (mechanistic Target Of Rapamycin Complex 1) phosphorylates 4E-binding proteins (4E-BPs) and inhibits their sequestering activity towards eIF4E, thus upregulating translation^6–8^.

In eukaryotic cells, a further mean of translation regulation consists in sorting mRNAs to different intracellular localization. According to their destination in the cells, mRNAs can indeed be sequestered from the ribosome machinery^9^ or have their translation enhanced^10^. This can be evident at both spatial and temporal levels. For instance, analysis of mRNA localization in *Drosophila* showed their different localization in specific cell compartments during embryonic development^11^. Interestingly, components of the translational machinery, namely eIF4E, eIF4A1, and also 4E-BP1, have also been localized to centrosomes^12–14^. These observations suggest a still unexplored link between the translational machinery and the centrosome.

*OFD1* encodes a centrosome/basal body-associated protein^15^ with a critical role in cilia formation^16–18^. Mutations in *OFD1* have been associated with Oral-facial-digital type I (OFDI) syndrome, a pleiotropic disorder characterized by renal cystic disease^19–21^. Other cilia-associated disorders with renal involvement include autosomal dominant (ADPKD1 and 2 associated to mutations in PKD1 and 2, respectively) and recessive renal cystic disease, Nephronophthisis (NPHP), Bardet-Biedl (BBS), as well as von Hippel-Lindau, Tuberous Sclerosis (TSC) syndromes^22^. Studies also link the RNA-binding protein bicaudal C homolog 1 (Bicc1) to renal cystic disease in patients and animal models^23^.

We now demonstrate that OFD1 interacts with PIC and eIF4F components and modulates Bicc1/eIFs interaction to functionally control the protein synthesis machinery in an mTORC1-independent manner. We also show *in vivo* that OFD1 controls the translation of specific mRNA targets in the kidney. Interestingly, both the eIFs and the mRNA targets were localized to centrosome. In addition, we demonstrate that OFD1 controls Bicc1 localization to the centrosome. Our findings suggest novel functions for the centrosome/basal body and provide new clues to follow on the molecular mechanisms underlying renal cystic disease.

## Results

### OFD1 interacts with the Translation machinery

**Figure 1:**
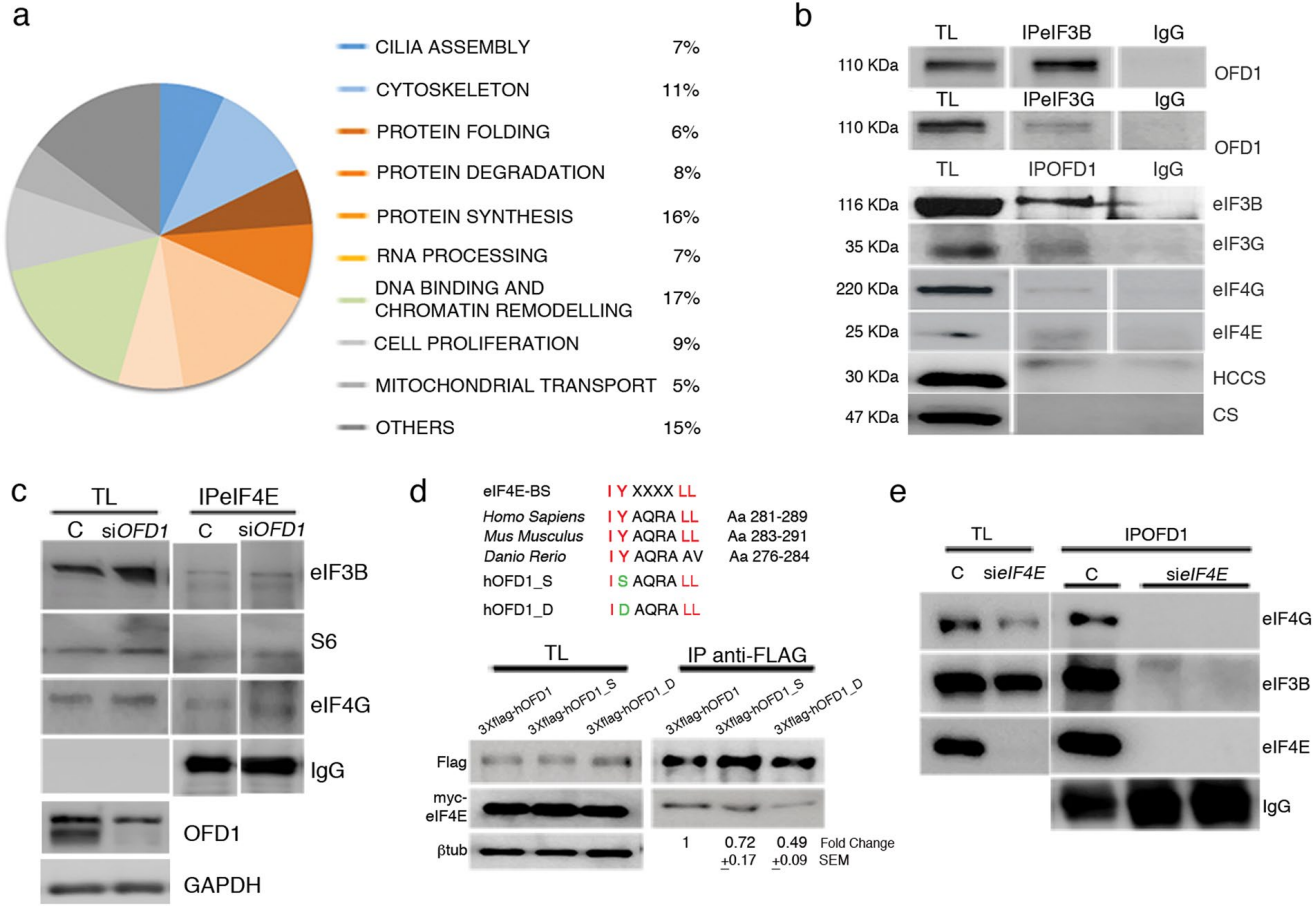
OFD1 interacts with the translational machinery. (**a**) OFD1 interactome. The putative OFD1 partners were clusterized in functional classes, which are identified by a color code. The majority of interactors are involved in the control of the cellular protein content (different shades of orange and red). (**b**) OFD1 interacts with components of the PIC and the eIF4F complex. Co-IP experiments performed on total lysates (TL) from HEK293 cells. (Upper panels) eIF3B and eIF3G were immunoprecipitated and OFD1 was detected by WB. (Lower panels) OFD1 was immunoprecipitated and different subunits of the PIC and of the eIF4F complex (eIF3B, eIF3G, eIF4G, eIF4E) were detected by WB. IgG were used as control. Holocytochrome-c type-synthase (HCCS) and Citrato synthase (CS) were used as negative controls. All lanes are from the same blot. However some of the lanes were not adjacent and are separated by a space. (**c**) Co-IP experiments demonstrate that eIF4E binds other PIC and eIF4F components (eIF3B, eIF4G and S6) in OFD1 silenced cells. Lower panel in c reports the effect of OFD1 silencing at the protein level. (**d**) OFD1 directly binds eIF4E. An eIF4E binding site (eIF4E-BS, Aa in red) is recognized in the OFD1 protein and conserved in OFD1 vertebrate homologs. Aa mutagenized within the eIF4E-BS are depicted in green. Co-Ip experiments demonstrated that the mutated constructs show a lower affinity for eIF4E (right panel). βtubulin was used as loading control. TLs are reported on the left. The eIF4E/OFD1 ratio was calculated and the fold change was reported below the panels as the mean ± standard error of the mean (SEM). (**e**) eIF4E mediates the interaction of OFD1 with eIFs. OFD1 was immunoprecipitated in control and *eIF4E*-silenced HEK293 cells and eIF4G, eIF3B and eIF4E were detected by WB. IgG were used as loading control.

A proteomic approach based on nano-LC-MS/MS analyses identified OFD1 putative interactors which included proteins involved in cellular processes such as cilia and cytoskeleton assembly, protein folding and degradation, RNA processing, DNA binding and chromatin remodeling. Sixteen per cent of the putative interactors corresponded to components of the protein synthesis machinery, such as ribosomal proteins and subunits G and B of the eIF3 complex, which is a PIC component (Fig. 1a and Supplementary Table S1).

The interaction between endogenous OFD1 and eIF3B, eIF3G, eIF4E and eIF4G was confirmed by co-immunoprecipitation (co-IP) experiments (Fig. 1b and e). We then asked whether OFD1 could modulate the formation of the PIC and/or of the eIF4F complex. Co-IP experiments demonstrate that eIFs interactions do occur and that the translational machinery is normally formed in the absence of OFD1 (Fig. 1c and Supplementary Fig. 2). Further analysis revealed the presence of an eIF4E-binding site (eIF4E-BS) highly conserved among OFD1 homologous proteins (Fig. 1d) in vertebrates. We mutagenized the eIF4E-BS conserved tyrosine-282 (Y) in serine (S), which belongs to the same class of amino acids (Aa) (polar), and in aspartate (D), a negatively charged Aa. Co-IP experiments demonstrated that OFD1 directly binds eIF4E, since mutations of the eIF4E binding site result in decreased affinity between endogenous OFD1 and the eIF4E constructs. This was more evident when Y282 was mutated in D (Fig. 1d). In addition, we silenced eIF4E in HEK293 cells and immunoprecipitated OFD1. In this condition, we observed that OFD1 loses its ability to bind other eIFs (Fig. 1e). Taken together these results suggest that OFD1 interacts with at least some components of the translational machinery and directly binds eIF4E, which in turn mediates PIC/eIF4F/OFD1 interaction.

### OFD1 controls Cap-dependent translation

**Figure 2:**
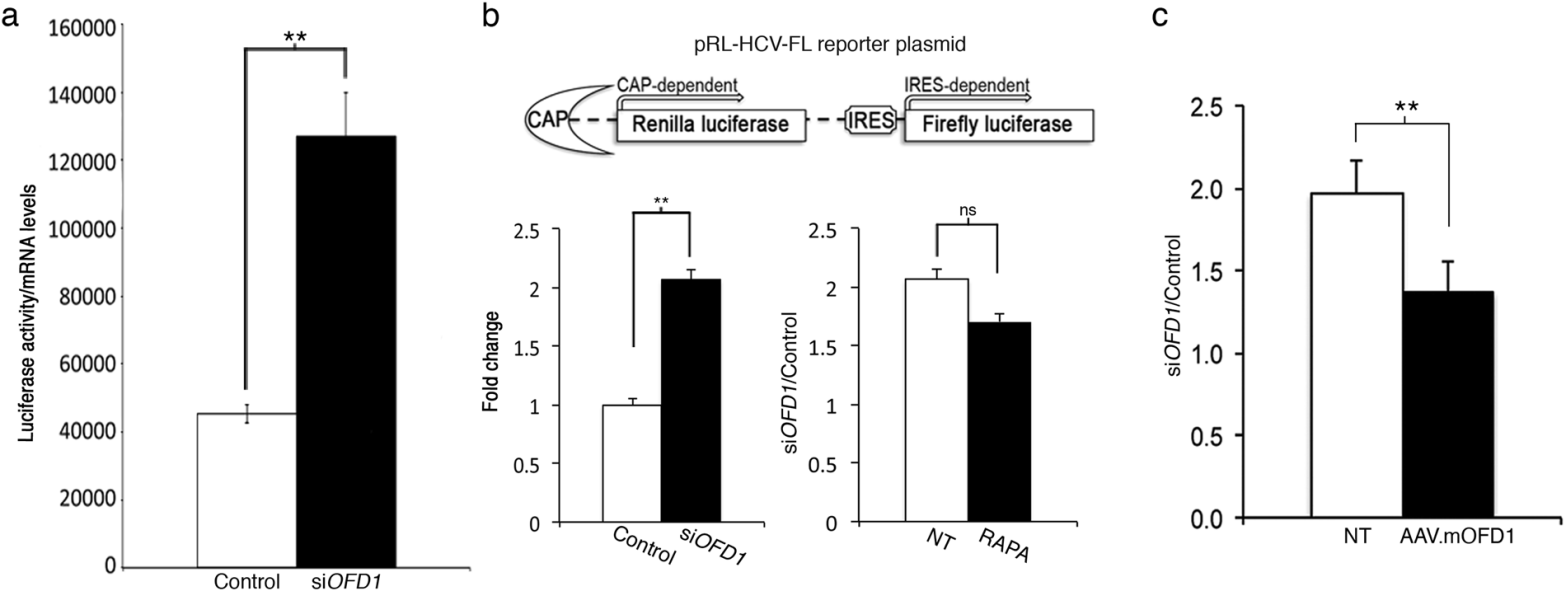
Cap-dependent translation is increased in *OFD1*-silenced cells. (**a**) *OFD1*-silenced cells (si*OFD1*; black bar) and control cells (Control; white bar) were transfected in triplicate with a plasmid overexpressing the Renilla luciferase under a constitutive promoter. Luciferase activity and *Renilla* mRNA levels were calculated and the ratio was reported in the graph. (**b**) On the top a scheme of the pRL-HCV-FL reporter plasmid is depicted. *OFD1*-silenced cells (si*OFD1*; black bar) and controls (Control; white bar) were transfected in triplicate with the reporter plasmid. The Renilla/Firefly luciferase light-unit ratio was calculated; the value for control cells was set at 1 and the fold change for all the samples was calculated and reported in the graph (left). The same experiment was performed in cells treated with rapamycin (RAPA, black bar) (graph on the right). The *OFD1*-silenced (si*OFD1*)/control (Control) cells ratio was calculated in untreated (NT, white bar) and treated (black bar) cells. (**c**) The accumulation of Renilla was rescued by overexpressing the murine form of Ofd1 (AAV.m*Ofd1*), which is insensitive to siRNA. Data are presented as the mean ± SEM. Student’s t-test or wilcoxon test was used to calculate the p value as reported in Methods. **p-value < 0.02; ns: p-value > 0.05.

*OFD1*-silenced and control HEK293 cells were transfected with a construct overexpressing the Renilla luciferase under a constitutive (HSV-TK) promoter. Renilla mRNA levels were evaluated by Real-Time PCR and were comparable in the two systems (Supplementary Fig. 1a). We then measured luciferase activity and calculated the protein/RNA ratio. This ratio was higher in *OFD1*-silenced cells compared to controls, suggesting that OFD1 acts as a negative regulator of translation (Fig. 2a).

It is well established that the formation of the PIC and eIF4F complex drive translation of capped mRNAs^1, 2^. To test whether OFD1 was involved in the regulation of Cap-dependent translation we transfected *OFD1*-silenced and control HEK293 cells with the pRL-HCV-FL bicistronic reporter plasmid^24^. In this construct the levels of Renilla luciferase represent Cap-driven translation efficiency. Firefly luciferase is under HCV-IRES regulation and is Cap-independent and therefore was used as a control reporter (Fig. 2b, top panel). This assay allowed us to demonstrate that the Renilla/Firefly luciferase ratio was higher in *OFD1*-silenced cells compared to controls (Fig. 2b, left) indicating that OFD1 specifically regulates Cap-dependent translation.

We previously demonstrated the upregulation of phosphorylated S6 ribosomal protein (rpS6), readout of mTORC1 activity, in *OFD1*-depleted models^25^. To test whether mTOR pathway contributes to the increased translation efficiency of Renilla luciferase, we transfected the pRL-HCV-FL reporter plasmid in the presence of rapamycin, a negative regulator of mTORC1 and consequently of Cap-dependent translation^26^. Rapamycin treatment resulted, as expected, in decreased levels of phosphorylated rpS6 (indicated in the figures as PS6) in both control and *OFD1*-silenced cells (Supplementary Fig. 1b). Rapamycin treatment resulted in an inhibition of ~30% of translation in both si*OFD1* treated and control cells (Ref. 24 and Supplementary Fig. 1b). The increase in the amount of Renilla luciferase in *OFD1*-silenced cells was only partially reverted by rapamycin treatment (Fig. 2b). This suggests an accumulation of the exogenous protein prior the drug administration. We validated the effect of OFD1 inactivation on Cap-dependent translation by transfecting the pRL-HCV-FL construct in HeLa cells. We show that OFD1 depletion does not result in perturbation of mTORC1-dependent phosphorylation of rpS6 (Supplementary Fig. 1c,d). Collectively, these results suggest that the role of OFD1 in protein synthesis is mTOR-independent.

We also treated HEK293 cells with cycloheximide (CHX), an inhibitor of translation. After CHX treatment, the rate of degradation of Renilla was comparable between *OFD1*-silenced and control cells, suggesting that normal protein degradation occurred and that the accumulation of Renilla observed in *OFD1*-silenced cells was due to upregulation of translation (Supplementary Fig. 1e). To confirm the specificity of the siRNA against human OFD1 we overexpressed the murine Ofd1, which is not silenced by the siRNA (Supplementary Fig. 2) against the human transcript and we obtained an almost complete rescue of luciferase accumulation in *OFD1*-silenced cells (Fig. 2c) thus supporting the role of Ofd1 in translation.

### OFD1 controls the protein synthesis of specific targets in the kidney

To verify the biological relevance and the physiological significance of our findings, we set up experiments to study the role of Ofd1 in translation *in vivo* in a conditional mouse model (*Ofd1*-IND) in which the *Ofd1* gene is inactivated at Postnatal day (P)0^27^. *Ofd1*-IND mice display about 80% Ofd1 inactivation. Renal tubule dilation appeared at P10, while at P18 the majority of the renal parenchyma was replaced by cysts. Biochemical and immunofluorescence (IF) studies revealed increased levels of phosphorylated rpS6 and 4E-BPs, two of the main targets of the mTORC1 pathway starting from P10 (Supplementary Fig. 3).

**Figure 3:**
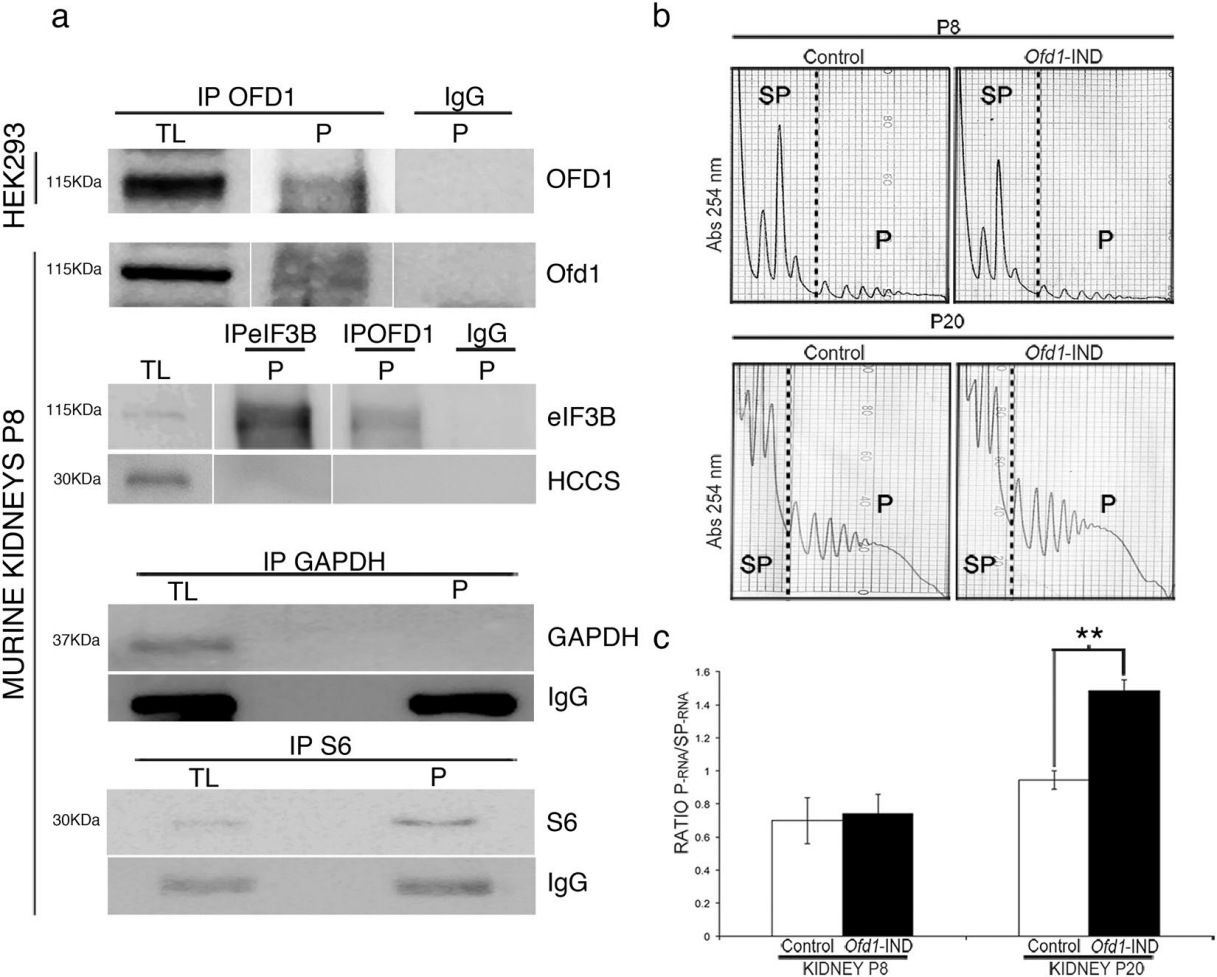
OFD1 is present in polysomal fractions and regulates the translation of specific targets in the kidney. (**a**) OFD1 coimmunoprecipitates with eIF3B *in vivo* in polysomes. We immunoprecipitated the OFD1 protein in TL (first lane) and in polysomal fractions (P, second lane) both in HEK293 cells (top panel) and in wt kidney extracts (P8) (lower panels) and the OFD1 protein was detected by WB. In TL the abundance of immunoprecipitated OFD1 probably reflects the localization of the protein in different cell compartments compared to the P fraction. Co-IP experiments confirmed that the interaction between OFD1 and eIF3B occurs also *in vivo* in polysomal fractions. eIF3B and rpS6 were immunoprecipitated from P fractions. Bands appeared enriched. These markers were used to demonstrate specificity of the P fractions. GAPDH was detected by immunoprecipitation in TL from kidneys and was absent in P and was used as a control for specificity. HCCS was used as a negative control both for the specificity of polysomal fractions and of the Co-IP. All lanes are from the same blot. However some of the lanes were not adjacent and are separated by a space. (**b**) Polysomal profiles observed in kidneys from *Ofd1-*IND mutants at P8 (upper panels) and P20 (lower panels). The peaks show the absorbance (Abs 254 nm) measured in the collected fractions. The polysomal (P) and subpolysomal (SP) areas are indicated. The polysomal fractions were obtained from two independent experiments for each sample. (**c**) Analysis of the polysomal profile. The analysis was performed on renal extracts from *Ofd1-*IND mice calculating the ratio between Polysomal RNA (P-RNA) and subpolysomal RNA (SP-RNA) in Controls (white bars) and *Ofd1*-IND mice (black bars). The RNA was obtained from kidneys of *Ofd1-*IND mutants and Controls at P8 (precystic stage) and at P20 (cystic stage). Data are presented as the mean ± SEM. Student’s t-test was used to calculate the p-value. **p-value < 0,05 at P20.

Polysomes represent a complex of ribosomes bound to mRNAs that are being actively translated. These mRNAs can be separated from untranslated mRNA by extracting polysomal fractions^28^. We extracted polysomes from HEK293 cells and wild-type (wt) murine kidneys and demonstrated the presence of the OFD1 protein in polysomes at P8 and P20 (Fig. 3a and data not shown). We also validated *in vivo* the interaction between Ofd1 and eIF3B in polysomes extracted from wt kidneys (Fig. 3a).

We then analyzed the renal polysomal profile from *Ofd1*-IND mice (Fig. 3b). The polysomal RNA content was quantified *in vivo* at precystic (P8) and cystic (P20) stages. A significant difference was observed at P20 (Fig. 3c), correlating with rpS6 phosphorylation at this stage (Supplementary Fig. 3). At P8, when the levels of phosphorylated rpS6 are comparable between OFD1 depleted models and controls, (Ref. 25 and Supplementary Fig. 3), the polysomal profile displayed a similar pattern between *Ofd1*-IND and controls, indicating that mRNA translation, as a whole, is not altered (Fig. 3b,c). We reasoned that OFD1 might regulate the translation of specific targets *in vivo*. We then performed microarray analysis on total and polysomal mRNAs from controls and *Ofd1*-IND mutant kidneys. We choosed to perform the experiments at the precystic stage P8 to avoid conditions of mTORC1 activation, as marked by rpS6 phosphorylation. We identified 141 targets differentially present in polysomal mRNAs (p-value < 0.05) (Supplementary Table S2). Comparing *Ofd1*-IND to control samples, nine targets showed different levels in both total and polysomal RNA. This indicates a bias possibly due to transcription and/or mRNA stability, independently from translation efficiency. On these bases, we did not proceed with further characterization of those mRNAs. The remaining 132 targets were subjected to bioinformatics analysis (Supplementary Table S3). Gene Ontology of the differentially translated mRNAs did not reveal significant enrichment of biological terms or functions. Gene co-expression relationships provide important clues about gene function. We thus decided to verify whether the mRNAs were co-expressed and queried the Netview tool^29^ with the 132 murine probe sets (127 unique gene symbols) representing the differentially translated targets. Our analysis revealed a mouse sub-network containing 78 nodes and hierarchical clustering showed two separate clusters of correlated genes, namely Cluster-1 and -2 (Supplementary Fig. 4A and Supplementary Table S4). Similar results were observed by loading the mouse sub-network in Cytoscape^30^ and by performing the analysis on the entire collection of 141 targets (Supplementary Fig. 4B,C). Forty-nine out of the differentially translated transcripts were unrelated to the others. Over 95% of Cluster-1 transcripts were underrepresented in polysomes while all transcripts belonging to Cluster-2 were enriched (Supplementary Table S5).

### OFD1 targets accumulate in cystic kidneys

**Figure 4:**
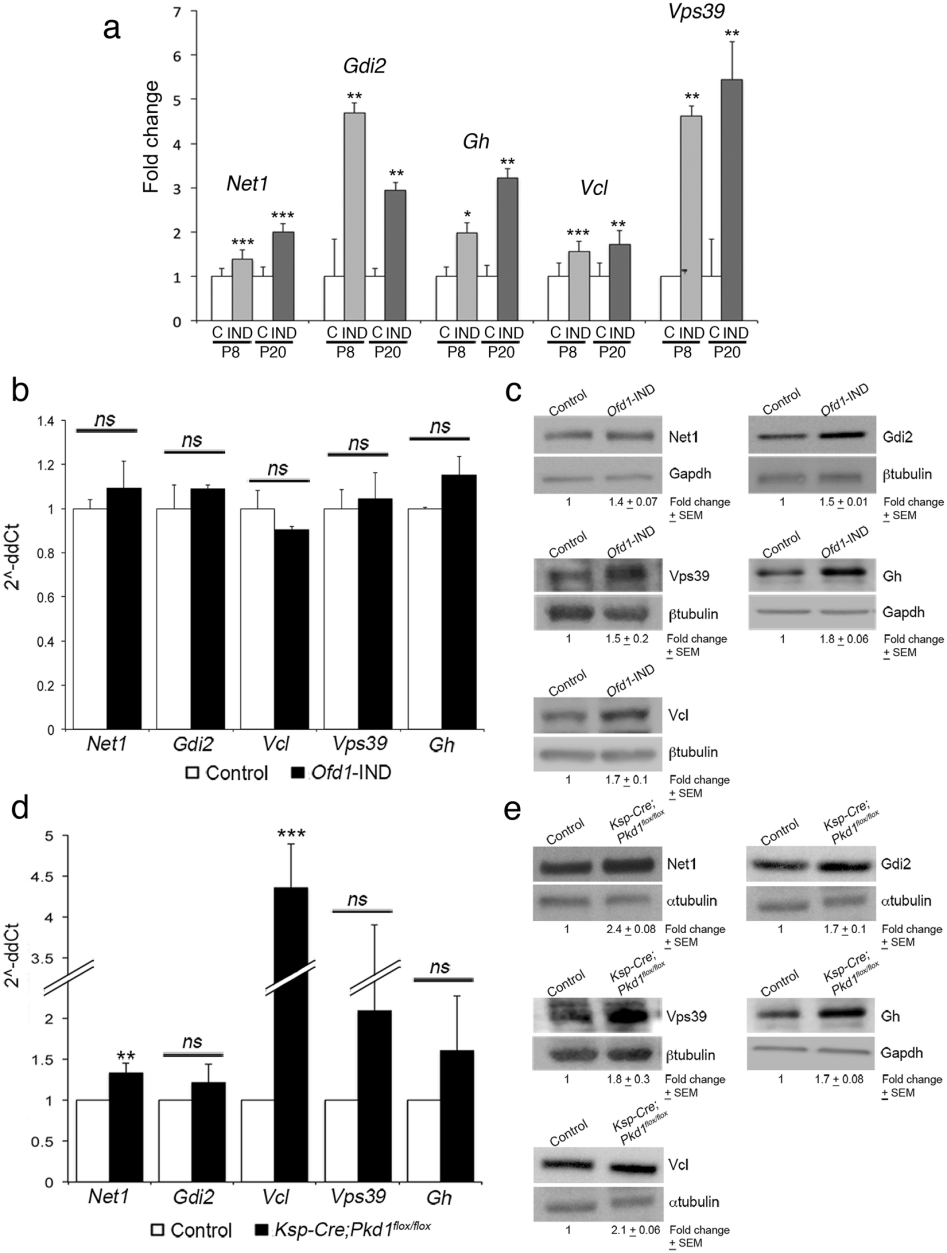
Accumulation of specific proteins in *Ofd1*-IND and *Ksp*-Cre;*Pkd1* ^*flox/flox*^ mutant kidneys. (**a**) Real Time-PCR on polysomal mRNAs. The enrichment of *Net1*, *Gdi2*, *Vcl*, *Vps39* and *Gh* was validated in kidneys of *Ofd1*-IND mutants (IND; gray bars at P8, black bars at P20) compared to controls (C; white bars) both at P8 and P20. (**b–d**) Transcriptional levels for *Net1*, *Gdi2*, *Vcl*, *Vps39* and *Gh* were measured by Real-Time PCR on mRNA extracted from total kidneys of controls (white bars) and *Ofd1*-IND mice (black bars in b) and *Ksp-Cre;Pkd1* ^*flox/flox*^ mutants (black bars in d). WB analysis showed the accumulation of all targets in renal lysates from both *Ofd1*-IND (**c**) and *Ksp-Cre;Pkd1* ^*flox/flox*^ mutants (**e**). For Gdi2 and Vcl (in e) the αtubulin used for normalization come from the same blot. Data are presented as the mean ± SEM. Student’s t-test was used to calculate the p-value. ns: not significant, p-value > 0.05; *p-value < 0.05; **p-value < 0.05; ***p-value < 0.01.

Since our *in vitro* data suggested that OFD1 is a negative regulator of translation, we focused on the characterization of the mRNAs enriched in polysomes upon Ofd1 depletion (Cluster-2). Previous data linked RhoA and the actin cytoskeleton to ciliogenesis^31, 32^, and renal cysts^33^ we thus selected for further validation, among the identified targets, transcripts that encode RhoA-actin associated proteins, namely Neuroepithelial-Cell-transforming-1 (*Net1*), vinculin (*Vcl*) and GDP-dissociation-inhibitor-2 (*Gdi2*). In addition, we selected the Vacuolar-Protein-Sorting-39 homolog (*Vps39*) as an example of a correlated target not associated to cilia biology and the Growth Hormone (*Gh*) as an unrelated target, not present in any cluster. We first performed Real-Time PCR on renal polysomal mRNAs extracted at P8 and P20 and confirmed increased translation of the five targets (Fig. 4a). We also measured mRNA total levels and demonstrated that they were comparable in *Ofd1*-IND mutants and controls (Fig. 4b). We then analyzed kidney lysates from *Ofd1*-IND mice and confirmed increased protein levels for all targets analyzed (Fig. 4c). We asked whether the accumulation of the targets is due to translation efficiency and not protein degradation impairment. *OFD1*-silenced and control cells were treated with cycloheximide (CHX), a potent inhibitor of protein synthesis, and with MG132, a known proteasome inhibitor. Analysis of NET1, GDI2 and VCL was not informative probably due to the half-life of the proteins which is unknown for NET1 and >30 hours for GDI2 and VCL^34^. Our results indicated that the accumulation of VPS39 and GH observed in *OFD1*-silenced cells was recovered only by CHX treatment suggesting that the proteins are normally degraded and that their accumulation is associated with increased synthesis (Supplementary Fig. 4d). We also tested two of the targets that were depleted from polysomes upon *Ofd1* inactivation, namely ACSL4 and CPT1A. We indeed demonstrated that their protein levels were reduced in *Ofd1*-IND samples (Supplementary Fig. 4e).

We also tested an additional model of inherited renal cystic disease, the *Ksp*-Cre;*Pkd1* ^flox/flox^ mouse, in which the *Pkd1* transcript, whose human homolog is mutated in ADPKD1, is conditionally inactivated in renal tubules resulting in renal cysts at birth^35^. Interestingly, we observed protein accumulation for all targets analyzed in kidney lysates from *Ksp*-Cre;*Pkd1* ^flox/flox^ mutants (Fig. 4e). In this model mRNA levels for *Vcl* and *Net1*were increased while no significant differences were observed for the other targets (Fig. 4d). These results demonstrated renal protein accumulation of all targets analyzed in two different murine models of inherited renal cystic disease.

### Bicc1 binds OFD1 target mRNAs

**Figure 5:**
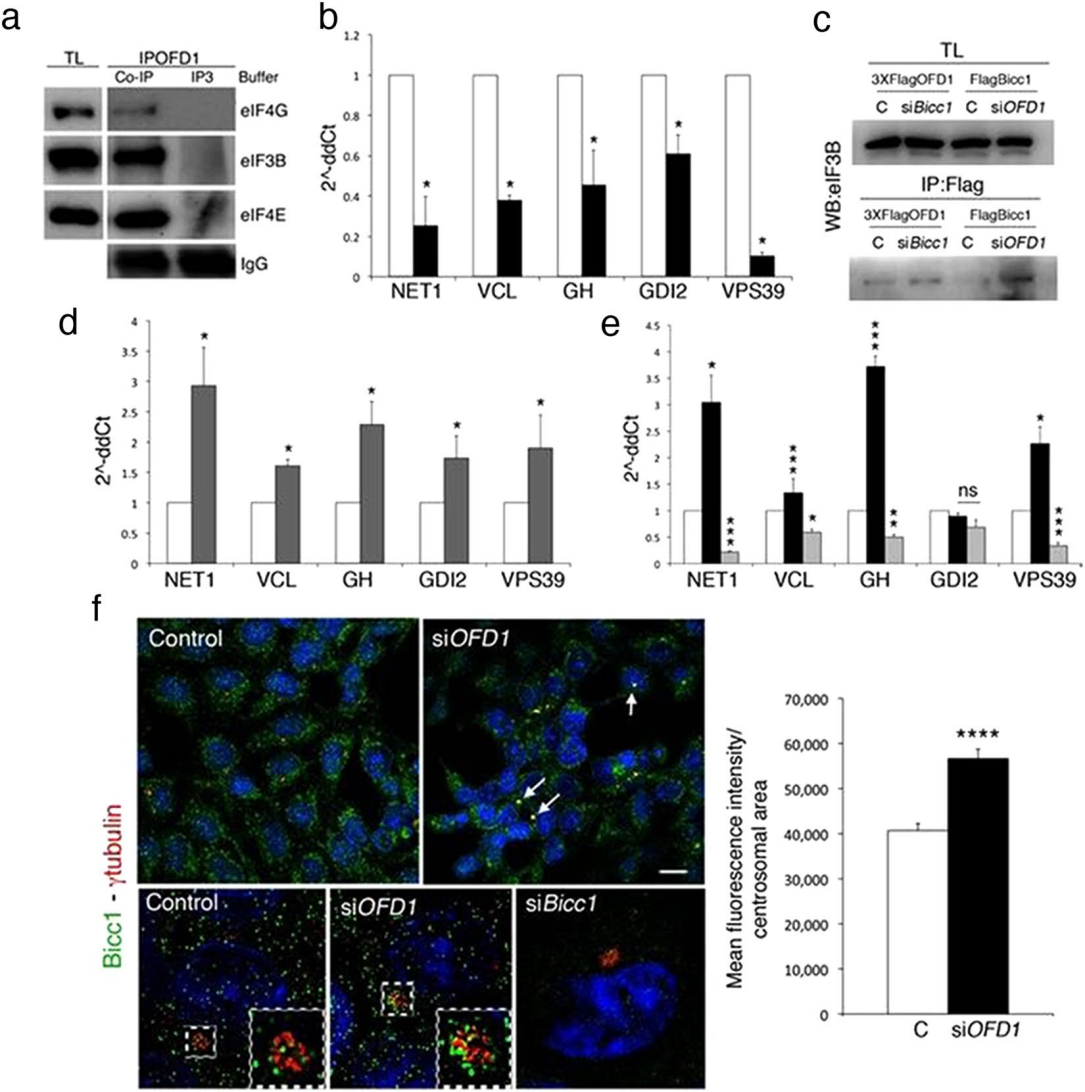
OFD1 cooperates with Bicc1 to control the translation of specific mRNAs. (**a**) Co-IP experiments demonstrate that the IP3 buffer destroys OFD1/eIFs interactions, which are preserved using the Co-IP buffer. (**b**) Real-Time PCR of the OFD1-bound mRNA shows that *Net1, Vcl, Gh, Gdi2 and Vps39* are not enriched after OFD1 IP (black bars) compared to Control (eIF4E-IP white bars). (**c**) Silencing of *Bicc1* results in stronger eIF3B/OFD1 affinity. Similar results are observed when *OFD1* is silenced and eIF3B/Bicc1 interaction is analyzed. (**d**) Real-Time PCR of the Bicc1-bound mRNA shows that mRNAs target are enriched after Bicc1 IP (grey bars) compared to Control (eIF4E-IP white bars). (**e**) RNA binding experiments are performed in *OFD1*- and *Bicc1*-silenced cells. In the absence of OFD1 the binding of target mRNAs to eIF4E (black bars) is more efficient compared to controls (white bars); while silencing of *Bicc1* results in decreased mRNA enrichment (grey bars). (**f**) IF with an antibody against Bicc1 (green) shows that Bicc1 colocalizes with γtubulin (red) at the centrosome and that the amount of centrosomal Bicc1 increases in *OFD1*-silenced cells (white arrows). Representative superresolution images are reported and a magnification of the centrosome is shown in the dotted white box. IF in *Bicc1*-silenced cells is reported as control for antibody specificity. Bicc1 localization at the centrosome was quantified by ImageJ and reported in a graph on the right. Main fluorescence intensity was calculated in the centrosomal area and reported in white for controls (C) and in black for *OFD1*-silenced cells (si*OFD1*). Data are presented as the mean + SEM. ns p-value > 0.05; *p-value < 0.05; ***p-value < 0.01 ****p-value < 0.005. Representative images were taken at the same contrast and reported. Bar = 5 μm.

Our results demonstrated that OFD1 is able to regulate the translation of specific mRNA targets. We first asked whether OFD1 could directly bind the specific mRNAs and set up an mRNA binding experiments. We immunoprecipitated 3XFLAG-OFD1 in a detergent and salt enriched buffer to disrupt OFD1/eIFs interactions (Fig. 5a). We then incubated the protein with total mRNA extracted from HEK293 wild type cells to avoid possible influences of OFD1 overexpression on transcription. As a control, an analogous experiment was performed by immunoprecipitating eIF4E in non-denaturating buffer to evaluate the levels of mRNA bound to the PIC/eIF4F as control. Real-Time PCR analysis on OFD1-bound RNAs revealed that the levels of NET1, VCL, GH, GDI2 and VPS39 were lower if compared to the PIC/eIF4F-bound mRNA, indicating that OFD1 alone does not efficiently bind these mRNAs (Fig. 5b). We then hypothesized that an mRNA binding protein could be responsible for the enrichment of mRNA targets in conditions of OFD1 depletion. Bicc1 is an mRNA transporter protein, that when mutated results in renal cysts development^36^. We thus looked at the presence of Bicc1 in the protein synthesis machinery by co-IP and found that overexpressed Bicc1 binds eIFs. Such interactions are stronger in *OFD1*-silenced cells while OFD1 still associate with eIF3B when Bicc1 is silenced (Fig. 5c). RNA-binding experiments followed by Real-Time PCR analysis demonstrated that NET1, VCL, GH, GDI2 and VPS39 are enriched after Bicc1 immunoprecipitation (Fig. 5d). To further validate our findings, we silenced *OFD1* and *Bicc1*, immunoprecipitated eIF4E in silenced and control cells in non-denaturating buffer and analyzed the target enrichment by Real-Time PCR. OFD1 silencing resulted in enrichment of the majority of targets, which are underrepresented after *Bicc1* silencing (Fig. 5e). Although we cannot exclude the presence of other cofactors, these results indicate that OFD1 and Bicc1 cooperate to modulate mRNA binding to eIF4E.

### Bicc1, the translational machinery and specific mRNAs colocalize to the centrosome

We performed immunofluorescence analysis and detected a diffuse cytoplasmic signal with a centrosomal localization for endogenous Bicc1 in control HEK293 cells. In OFD1 silenced cells, however, we observed a more abundant Bicc1/γtubulin colocalizing signal. To quantify this observation, we measured by ImageJ the intensity of the Bicc1 signal (green) in a space of 2 μm^3^ designed around the centrosomes and marked by γtubulin (Fig. 5f). These results indicate an enhanced recruitment of Bicc1 to the centrosome in conditions of OFD1 downregulation.

**Figure 6:**
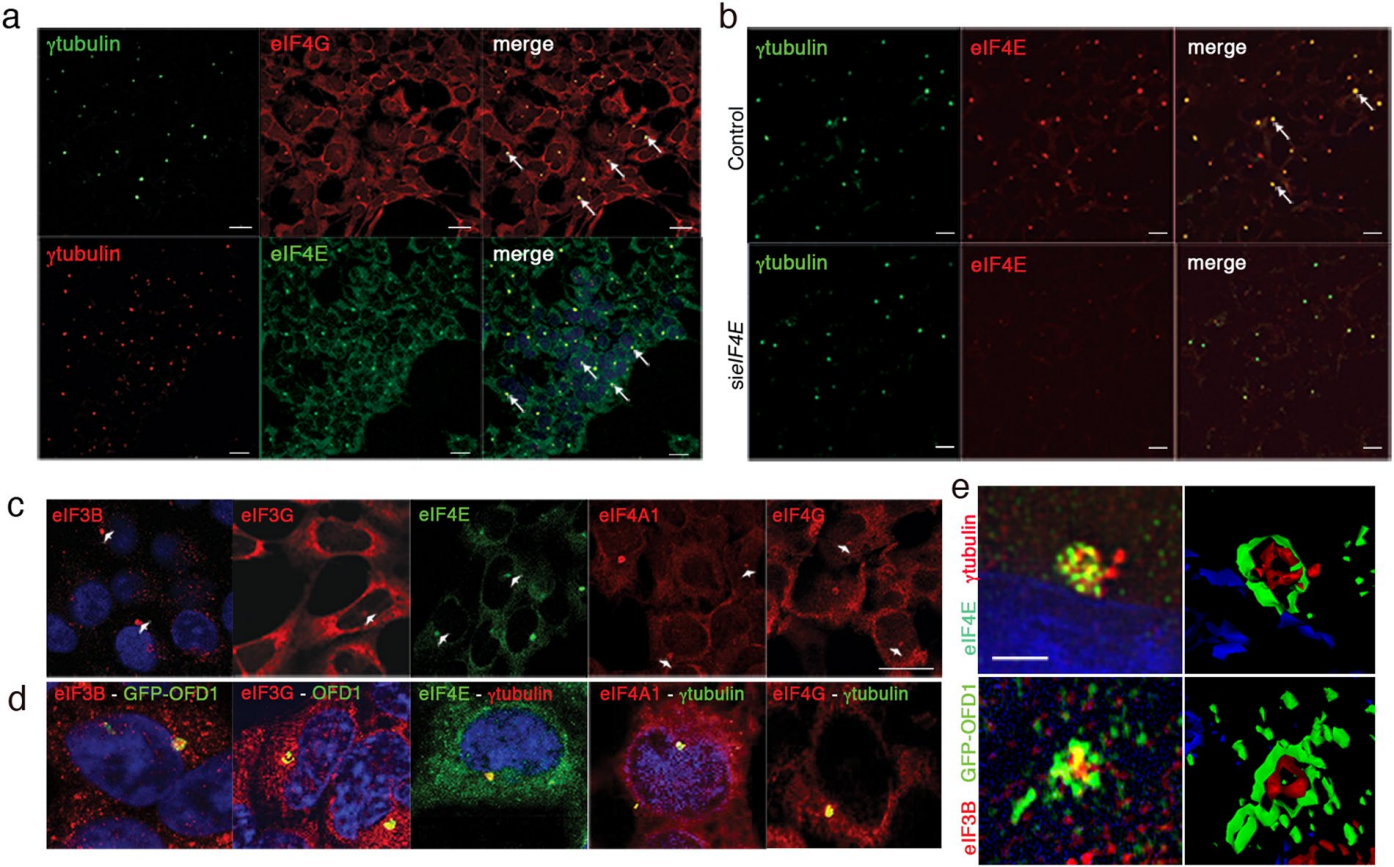
Translation initiation factors localize at the centrosome in HEK293 cells. (**a**) Immunofluorescence experiments demonstrate that eIF4G and eIF4E colocalize with γ, a centrosomal marker (arrows). (**b**) Silencing of *eIF4E* results in downregulation of the signal, confirming the specificity of the immunostaining of the eIF4E antibody. (**c**) Single channel IF are presented for each protein analysed, arrowheads indicate the staining that suggest the centrosomal localization. (**d**) Magnification of IF experiments showing colocalization of eIF3B, eIF3G, eIF4E, eIF4A1, eIF4G with centrosomal markers (OFD1 or γtubulin). (**e**) Analysis of centrosomal localization of eIF4E and eIF3B by Superresolution microscopy. IF (left panels) and 3D reconstruction (right panels) are provided. Bar = 10 μm. For Superresolution images Bar = 2 μm. For all panels *n* = 3.

Components of the translational machinery, namely eIF4E and eIF4A1, have been localized to centrosomes in non-mammalian systems^12–14, 37^. IF experiments in HEK293 cells demonstrated that eIF4E, eIF4A1 and eIF4G colocalize with γtubulin, a centrosomal marker (Fig. 6). In addition, OFD1 colocalizes at the centrosome with eIF3G and B (Fig. 6d,e). IF experiments performed on *eIF4E*-siRNA-treated cells demonstrated the specificity of the centrosomal eIF4E signal, which decreased in silenced cells (Fig. 6b).

**Figure 7:**
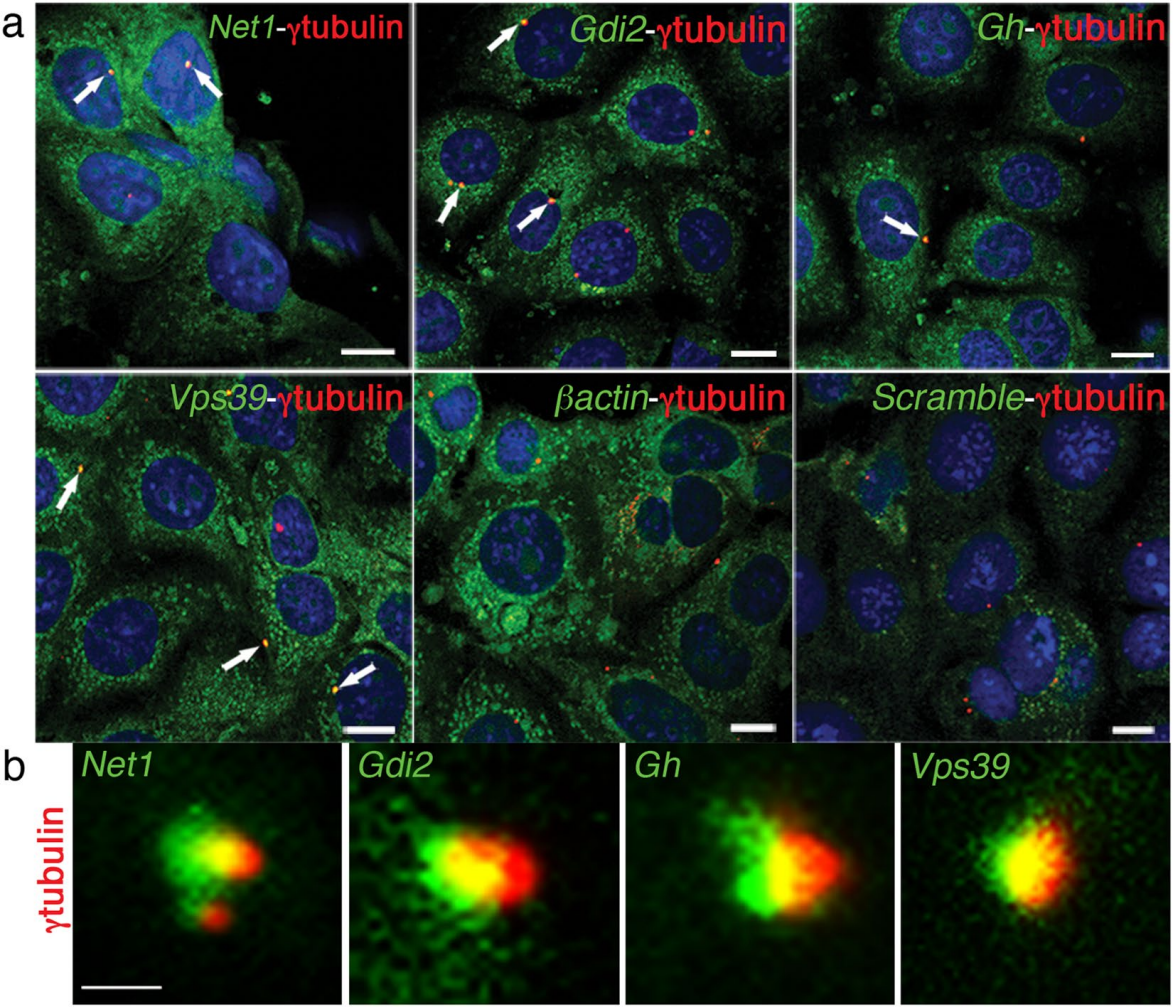
OFD1 target mRNAs localize at the centrosome. (**a**) *Net1, Gdi2, Gh* and *Vps39* mRNAs (green) colocalize with γtubulin (red) at the centrosome in IMCD3 cells (arrows indicate merged signals). βactin and scrambled probes (green) were used as controls. Bar = 10 μm. (**b**) Superresolution images show target mRNAs (green) and γtubulin (red) colocalization. Bar = 2 μm. Representative images were taken at the same contrast and reported.

We also evaluated the localization of specific mRNA targets. Renal cells were transfected with γtubulin-dsRed^38^ to label the centrosome/basal body. We then performed RNA *in situ* with locked nucleic acid (LNA) probes designed to recognize *Vps39*, *Net1*, *Gdi2* and *Gh* revealing a specific signal at the centrosome (Fig. 7 and Supplementary Fig. 5 and data not shown). The colocalization signal between *Vps39* and γtubulin was quantified and this analysis revealed that colocalization occurs in the majority of the cells. In addition, we observed that silencing of *Ofd1* resulted in an increased number of cells showing *Vps39* and γtubulin colocalization (Supplementary Fig. 5). A commercially available scrambled probe and β-actin were used as controls. All together, our data suggest that the centrosome could be a new station with a role in protein synthesis.

## Discussion

Our analysis of the OFD1 interactome revealed intriguing findings about this centrosome/basal-body protein. Some of the putative interactors are involved in expected functions such as cilia assembly and cytoskeleton organization, others in DNA binding and chromatin remodeling, in line with previous observations, demonstrating that OFD1 interacts with the TIP60 complex, which is involved in transcription and chromatin remodeling^15^.

The largest single category of putative interactors (37%) includes proteins linked to biological processes involved in the regulation of cellular protein content such as RNA processing, protein synthesis, protein folding and degradation. These findings are in line with previous observations linking cilioproteins to regulation of proteasomal activity, centrosome composition and mRNA processing^39–41^.

These results were functionally validated demonstrating that OFD1 directly binds PIC/eIF4F components and that the binding to eIF4E is necessary for PIC/eIF4F-OFD1 interaction. We showed that OFD1 specifically controls the translation of Cap-regulated mRNA reporters in human cells, *in vitro*. We then confirmed Ofd1 to have a role in regulating the translation of specific endogenous mRNAs *in vivo*, in mouse kidney.

Our RNA binding experiments indicate that OFD1 does not directly bind mRNAs. We found that Bicc1, an mRNA binding protein described in isolated cilia^42^, binds a subset of OFD1 mRNA targets. Interestingly, we show that OFD1 and Bicc1 modulate the binding of selected targets to eIF4E. This evidence suggests that the two proteins cooperate to regulate the translation of specific mRNAs. Bicc1 functions as repressor of protein synthesis through microRNA binding and cytoplasmic clustering^43, 44^. Our data suggest that this protein can also act as a positive regulator of protein synthesis. The role of Bicc1 in protein synthesis may differ according to the subcellular localization and the consequent availability of specific interactors/mRNAs. Moreover, we identified specific OFD1 translational targets: some of them resulted to be enriched and others depleted in renal polysomes. When transfecting an mRNA reporter in HEK293 cells, we found that OFD1 depletion is able to enhance translation at a more general level. This result may be explained by the mRNA overexpression, resulting in OFD1 and its cofactors exerting their function in translation independently from the specificity for their physiological targets. Overall, our results indicate that OFD1 could function both as negative and positive translation regulator. For instance, the impairment in the translation of specific mRNAs in presence of OFD1 on polysomes may be due to OFD1 sequestering pivotal translation factors. On the other hand, we found OFD1 association with the translation machinery to be modulated by Bicc1, suggesting that OFD1 interaction with the polysomes can be disrupted to increase translation efficiency. On the basis of *in vitro* and *in vivo* experiments, we here propose a role for OFD1 in regulating the translation of specific mRNAs.

We generated experimental evidence that suggest that OFD1, its target mRNAs and components of the translation machinery colocalize at the centrosome. Components of the translational machinery have been localized to centrosomes in Drosophila and yeast^37, 45^, although their role has not been fully understood. We now show a centrosomal localization of the translation machinery and of specific mRNAs in mammalian cells.

Eukaryotic cells spatially sort specific mRNAs to achieve mRNA translation directly where required in the cell^11^. Several examples of localized translation occurring also near cellular organelles have been described^46, 47^. Interestingly, Bicc1 has a role in controlling the spatial localization of mRNAs^43^. Our results also show that Bicc1 is recruited to the centrosome in absence of OFD1. On the basis of these results, we suggest that OFD1 could control the access of Bicc1/mRNAs to the translational machinery at the centrosome to functionally control protein synthesis. We propose that the centrosome/basal body could represent a specialized station to receive signals and rapidly modulate already known and yet to be determined specific functions of the nearby ciliary structures. Centrosomal translation could represent a mechanism by which cells respond, quickly and locally, to specific stimuli, as already described in neurons^48^. However, the molecular mechanism by which OFD1, Bicc1 and their possible cofactors impact translation needs to be fully characterized.

The OFD1 target mRNAs we identified are involved in different biological processes, e.g. cell death, mitochondrial biology, mRNA processing and metabolism. Recent data implicated defective metabolism in the pathogenesis of ADPKD^35^.

Interestingly, some of the identified targets (e.g. *Vps39*, *Arf6*, *Copb2*, *Gm4024*) were associated with vesicle-mediated transport. In 2013, Clement and colleagues demonstrated that clathrin-dependent endocytosis contributes to signal modulation at the pocket region of primary cilia^49^.

A subset of targets, namely *Net1*, *Gdi2* and *Vcl*, points to actin and focal adhesion dynamics which have been functionally related to cilia assembly^31, 32, 50^ and to the development of renal cysts^33^. Other targets, such as *Vps39* and *Gh*, belong to gene ontology categories not associated with cilia biology. However, GH secretion has been recently associated with the development of simple renal cysts in patients with acromegaly^51^. We validated accumulation of these five targets in two different mouse models of renal cystic disease (i.e. OFD1 and ADPKD). The remaining uncharacterized mRNAs may represent potential targets to be investigated for a putative role in renal cyst development.

Although post-transcriptional regulation of mRNA has not been clearly associated with renal cysts, it is noteworthy that Bicc1, which when mutated results in renal cystic disease and ciliary defects^52^, controls the stability of Pkd2 mRNA and its translation efficiency^53^. Future studies will clarify the potential involvement of post-transcriptional RNA regulation in renal cyst development.

mTORC1 is a positive regulator of translation^8^ and experimental data suggest a potential reciprocal relationship between cilia and the mTOR pathway^54, 55^. Deregulation of the mTOR pathway and ciliary dysfunction are often observed in renal cystic disease, although the functional link among mTOR, cilia and cysts is yet to be determined^56^. We previously demonstrated in Ofd1 mutants that deregulation of mTORC1 signaling is also evident in non-dilated renal tubules where cilia appear to be present, suggesting that the role of OFD1 in ciliogenesis is not related to mTORC1 activation^25^. We now have evidence pointing to OFD1 regulating protein synthesis independently from mTORC1. This is clearly shown by a) *in vitro* modulation of mTORC1; b) the presence of differentially expressed targets in polysomes extracted at P8 when the levels of rpS6 phosphorylation are not increased; and c) the finding of 72 transcripts depleted from polysomes. Moreover, the limited number of targets identified suggests that in physiological conditions OFD1 controls the translation only of specific mRNAs.

Some of the targets we identified, namely GH and Vps39, activate mTORC1^57, 58^ and their accumulation might underlie mTOR activation in OFD1 depleted models (Ref. 25 and Supplementary Fig. 3).

Translation components are localized throughout the cytoplasm. However, we demonstrate that: a) components of the translation machinery localize to the centrosome in mammalian cells; b) the centrosomal protein OFD1 physically interacts with proteins involved in translation regulation; c) OFD1 cooperates with the mRNA binding protein Bicc1, which is also involved in renal cystic disease, to functionally control the translation of specific mRNA targets.

To the best of our knowledge, OFD1 is the first example of a centrosomal protein directly involved in the regulation of translation. Our results highlight a possible role for centrosomal/basal body proteins in protein translation and provide functional clues for a better understanding of renal cystic disease.

## Methods

### Cell lines and cell treatments

Human Embryonic Kidney (HEK293) and Human cervical carcinoma (HeLa) cells were cultured in DMEM 10% fetal bovine serum (FBS). Murine inner medullary collecting duct (IMCD3) cells were cultured in DMEM-F12, 10% Fetal Calf Serum for RNA *in situ* hybridization experiments. Human Kidney 2 (HK2) cell were cultured in D-MEM/D-MEM F12 (1:1) 5% FBS supplied with 1% Glutamine and ITS (Insuline 5 ug/ml, Transferrine 5 ug/ml and Selenium 5 ng/ml) from SIGMA.

Media were supplemented with 100 Units/ml penicillin, and 100 μg/ml streptomycin. Cells were grown at 37 °C with 5% CO_2_.

Cycloheximide (CHX) (C-7698, SIGMA) and MG132 (C221, SIGMA) were used at 100 µM and 30 µM concentration, respectively, to treat cells for 6 hours.

### Proteomic studies

Lysis Buffer: 50 mM Tris-HCl pH 7.4, 150 mM NaCl, 1% Triton X-100, 0.1% Tween 20, 5 mM MgCl_2_, 10% glycerol, proteinase inhibitors.

Washing Buffer: 50 mM Tris-HCl pH 7.4, 300 mM NaCl, 1% Triton X-100, 0.1% Tween 20, 5 mM MgCl_2_, 10% glycerol, proteinase inhibitors.

HEK293 cells expressing 3XFLAG-OFD1 and the empty vector used as control were lysate with the Lysis Buffer. Total protein extracts were pre-cleared with mouse IgG agarose beads and incubated ON at 4 °C with M2 anti-FLAG agarose-conjugated antibody beads (Sigma). Non-retained proteins were then incubated with M2 anti-FLAG agarose-conjugated antibody beads (Sigma) overnight at 4 °C. Beads were washed with Washing Buffer. Retained protein complexes were eluted with 3XFLAG peptide, precipitated with methanol/chloroform and loaded on 10% polyacrylamide SDS-PAGE. Protein bands, stained with Coomassie colloidal blue (Pierce) were excised from gel and subjected to proteomic procedure (Supplementary Fig. 6). The control experiment obtained by immunoprecipitation of empty vector transfected cells with anti-FLAG agarose beads allowed to rule out unspecific retained proteins as described^59^.

Nanoscale liquid chromatography coupled to tandem mass spectrometry (nanoLC-MS/MS) analyses of peptide mixtures were performed on a CHIP MS Ion Trap XCT Ultra equipped with 1100 HPLC system and chip cube (Agilent Technologies, Palo Alto, CA, USA). After loading, the peptide mixture (10 µl in 0.2% formic acid) was concentrated and washed at 4 µl/min in the enrichment column (Agilent Technologies chip), with 0.1% formic acid. The sample was fractionated on a C_18_ reverse-phase capillary column onto the CHIP at a flow rate of 200 nl/min, with a linear gradient of eluent B (0.2% formic acid in 95% acetonitrile) in A (0.2% formic acid in 2% acetonitrile) from 7% to 60% in 50 min. Peptide analysis was performed using data-dependent acquisition of one MS scan (mass range from 400 to 2000 m/z) followed by MS/MS scans of the three most abundant ions in each MS scan. Raw data from nanoLC-MS/MS analyses were introduced into MASCOT software package version 2.4 (Matrix Science, Boston, USA) to search the NCBI human non-redundant protein database (NCBInr at www.matrixscience.com). NanoLC-MS/MS data were searched using a mass tolerance value of 600 ppm for precursor ions and 0.6 Da for MS/MS fragments, trypsin as the proteolytic enzyme, missed cleavages maximum value of 1, and Cys carbamidomethylation, pyroglutamate (peptide N-terminal Gln) and Met oxidation as fixed and variable modifications, respectively. Candidates with at least 2 assigned peptides with an individual MASCOT score > 18 were considered significant for identification.

### Constructs

Constructs overexpressing the OFD1 protein and the HEK293 stable clones used were described^15^. The AAV mOFD1 was obtained cloning the murine Ofd1 cDNA in pAAV2.1 CMV vector. Gerd Waltz provided the FLAG-Bicc1 construct (pcDNA6). Mutagenesis of p3XFLAGOFD1 was obtained using QuikChangeII kit (Agilent technology 200523). Primers for mutagenesis are displayed in Table S6. The pRL-HCV-FL bicistronic reporter plasmid was described^24^. pRL-TK Vector for Renilla luciferase was from Promega (E2241).

### Transfections

HeLa and HEK293 cells were transfected using TransIT®-LT1 Transfection Reagent (Mirus) according to the manufacturer’s instructions and cells were collected 72 h from transfection both for WB and IF. IMCD3 cells were transfected to overexpress γtubulin-dsRed^38^ with Lipofectamine2000 (Life technologies, 12566014) for RNA *in situ* hybridization experiments. As control, cells were treated with the Transfection reagent alone.

### Antibodies

The anti-OFD1 and anti-Ofd1, were generated against the human full-length OFD1 protein (NM_003611) and a portion of the murine Ofd1 homologous protein (NM_177429 Aa 461–884), respectively, and were previously described^60^. All other antibodies used in this study are commercially available and are listed below. From Santa Cruz Biotechnology: eIF3G (R-20 sc-16362), eIF3B (H-300 sc-28857), GAPDH (6C5 sc-32233), GH (sc-10364), VPS39 (D15 sc-104759), NET1 (H-70 sc50392), IgG mouse (sc-2025), IgG rabbit (sc-2027). From Cell Signaling Technology: eIF4E (9742), eIF4G (2498), phosphorylated rpS6 (Ser240/244, 5364), eIF4A1 (2490), phosphorylated 4E-BP1 (Thr37/46, 9459). From Sigma-Aldrich: VCL (clone hVIN-1 V9131), γtubulin (clone GTU-88 T6557) and acetylated tubulin (T6793). From Spring Bioscience GDI2 (D4-GDI E2430). References for antibodies’ specificity^14, 61^: for eIF4E and eIF4A1^62^; for eIF3G^63, 64^; for eIF3B.

### Immunoprecipitation (IP)

The IP were performed on HEK293 lysates cells at least three times. Buffer IP3 (see also Supplementary Fig. 5): 50 mM Tris-HCl pH 8, 150 mM NaCl, 1% Triton X-100, 1% Tween 20, 0.5% sodium deoxycholate, 10 mM MgCl_2_, 10% glycerol, protease inhibitors.

### Co-immunoprecipitation (co-IP) experiments and Immunoblot analysis

HEK 293 cells and kidneys from fed animals were homogenized in lysis buffer (Tris 50 mM pH 7.9, 1% triton X-100, 0.1% Tween20, 150 mM NaCl, 10% glycerol, 5 mM MgCl2) or in a specific buffer for Co-IP experiments (50 mM Tris-HCl, 1 mM EDTA, 10 mM MgCl_2_, 5 mM EGTA, 0.5% Triton X-100, pH 7.28). For Co-IP experiments, lysates were incubated with specific antibodies and IgG, as control, as described^65^. Co-IP experiments were performed at least three times. Western blot (WB) studies were performed at least in triplicate and representative images were shown. For Co-IP experiments the ratio of IP proteins with respect to the input was 1:50. Blots were quantified by ImageJ. The control was settled as 1 and the fold change was calculated and reported below the panels as the mean ± standard error of the mean (SEM). Animals were perfused with PBS to eliminate blood traces for detection of the GH protein. Lysates were treated with protease inhibitors from Sigma-Aldrich (P8340) and phosphatase inhibitors from Roche (PhosSTOP, 04906837001). Polyvinylidene difluoride (PVDF) membranes were used for Immunoblot (Millipore, US, Immobilon-P, IPVH00010) and ECL western blotting reagent (Thermoscientific, 32106) or Femto (Thermoscientific, 34095) were used for detection.

### Immunofluorescence (IF)

Cells were fixed in methanol or in PFA 4%. Blocking was performed in PBS 0.2% TritonX-100, 10% FBS. IF experiments were performed at least three times. For analysis of IF data more than 100 cells were counted for each experiment. The significance of the results was calculated by Student’s t-test and reported as pvalue. In IF experiments, co-localization at the centrosome were considered biological relevant when present in >50% of cells.

For the colocalization analysis at the centrosome, we selected the centrosomal area defined by γtubulin signal and measured the fluorescence signal intensity of the protein/mRNA of interest. An equal area was selected in three different positions in the cell and the average value was calculated and considered as the mean fluorescence intensity of the cell. Cells in which the signals were considered to colocalize were characterized by a higher fluorescence signal at the centrosome compared to the mean fluorescence intensity of the cell. Fluorescence intensity was calculated by ImageJ.

High-resolution confocal microscopy “LSM 880/Elyra PS-1” Zeiss with superresolution structured illumination processing was used to obtain high-resolution images.

### RNAi

ON-TARGET plus smart pool siRNAs against human *OFD1* (L009300000020), *eIF4E* (L00388400001010) and Nontargeting control pool (D00181010) from Darmachon were used at a concentration of 100 μM. The transfection reagent was INTERFERIN (409–10, Polyplus) or Lipofectamine RNAimax (1377800, Life Technologies). Silenced cells were used for both WB and IF analyses after 72 hours from transfections.

### RT-PCR and Real-Time PCR

Total RNA from cells and kidneys was extracted by the RNeasy Mini Kit from QIAGEN (74106). The SuperScript®III First-Strand kit by Life technologies (18080–051) was used according to the supplier’s protocol. The LightCycler® 480 SYBR Green I Master Mix (04707516001) was used for all samples. For quantitative PCR the final concentration of primers was of 0.4 μM on total extracts and 0.8 μM on Polysomal extracts and cDNA obtained from RNA binding experiments. The ΔΔCT method was used for statistical analysis to determine gene expression levels. Primers that amplify the *Gapdh* transcript were used as internal reference. All experiments contained three technical replicates and were performed at least three times. Primers for RT and Real-Time experiments were reported in Supplementary Table S6.

### Bicistronic Luciferase assay

For luciferase reporter experiments, HEK293 cells were transfected with the pRL-HCV-FL reporter plasmid. Forty-eight hours post-transfection the luciferase activity was measured using the Dual-Luciferase Reporter Assay System (Promega) and Glomax96 microplate luminometer (Promega) according to manufacturers’ instructions. Rapamycin 100 nM (AY-22989, LC Laboratories) and Cycloheximide 100 μΜ (C-7698, SIGMA), were used to treat cells for 5 hours. All assays contained three technical replicates and were performed at least three times. Normal distribution of values was evaluated with the Shapiro test. To calculate the p-value we used the Student’s t-test for normal distributions and the Wilcoxon test when samples were not normally distributed. The standard error of the mean (SEM) was calculated for each experiment as the SEM of a simple mean or mean of ratio.

### Polysome fractionation and polysomal RNA extraction

Hypotonic buffer: 5 mM Tris-HCl pH 7.5, 1.5 mM KCl, 2.5 mM MgCl_2_.

Extraction Buffer: hypotonic buffer, 0.5% triton X-100, 0.5% Na-deoxycholate, 120 U/ml RNAse inhibitors [AM2692 from Ambion], 3 mM DTT, 100 μg/ml cycloheximide.

Buffer3: 50 mM Tris-HCl pH 7.8, 240 mM KCl, 10 mM MgCl_2_, 250 mM sucrose.

Solution D: 4 M Guanidinium thiocyanate, 25 mM Na-Citrate pH 7, 0.5% Sarcosyl, 100 mM 2-MeSH Mercaptoethanol).

HEK293 cells: Cells were treated for 10 min with 100 μg/ml cycloheximide (C-7698 from Sigma-Aldrich) and washed with Hypotonic Buffer. Cells were lysed in Extraction Buffer and, after quantification, 1 mg/ml of heparin was added.

Kidneys: Tissues were lysed in 1 ml of Buffer3 with 5 mM DTT, 100 μg/ml cycloheximide, 2% triton X-100, 100 U/ml RNAse inhibitors and, after quantification, 1 mg/ml of heparin was added.

Equal amounts of cellular and kidney lysates were layered on 0.5–1.5 M linear sucrose gradient. Absorbance at 260 nm was registered in a curve. The area below the curve of subpolysomal (SP) and polysomal (P) fractions was calculated using the Adobe Photoshop program and the SP/P ratio was calculated as read-out of general translation.

To purify the RNA, we added 1 ml of isopropanol to each fraction and put the mixed fractions at −20 °C ON. After 16 hours, the fractions were centrifuged for 30 min at 15000 rpm at 4 °C. Pellets were resuspended in SolutionD. Methods for polysome fractionation, polysomal RNA extraction and analysis of polysomal profile were described^28^.

Polysomal fractions from cells and Ofd1 mutant kidneys and controls were obtained from two different control and mutant animals from different littermates for each set of experiment.

### Animal Models

*Ofd1* ^fl/+^ females^16^ were crossed with pCAGGCre-ER^TM^ mice as described^27^. *Ksp-Cre;Pkd1* ^*flox/flox*^ mice were described^35^. *Cre* negative *Ofd1* ^fl/y^ and *Pkd1* ^flox/flox^ mice were used as control. All studies were conducted in strict accordance with the institutional guidelines for animal research and approved by the Italian Ministry of Health in accordance to the law on animal experimentation. All animal treatments were reviewed and approved in advance by the Ethics Committee of the Animal House facility of the Cardarelli Hospital, (Naples, Italy) (protocol number: 870/2015-PR; approval date August 24, 2015) and of the San Raffaele Scientific Institute (IACUC-548).

### Microarray experiments

For microarray analysis we collected polysomal and total RNA from kidneys of *Ofd1*-IND and WT mice at P8. We used the Affymetrix Mouse 430 A 2.0 array, 3′-IVT array. Microarray data were deposited on ArrayExpress_(E-MTAB-2827).

### RNA *in situ* hybridisation

Washing buffer 20xSCC: 175.2 g NaCl + 88.4 g sodium citrate in 1 L DEPC-H_2_O. Denhardt mix: 1 g BSA, 1 g Ficoll 400, 1 g polyvinylpurrolidon in 50 ml DEPC-H_2_O. Hybridisation mix: 50% formamide, 10% tRNA, 2% 50x Denhardt mix, 50% Dextran and 20% 20xSSC. Slides were washed with PBS, incubated with 0.1 M triethanolamine with 185 μl acetic anhydride for 10 min and washed with 2xSCC for 5 min RT followed by a 5 min wash in 2xSCC with formamide at 37 °C. Cells were permeabilised for 2 h at 54 °C with 0.1% tween in PBS for 10 min at 37 °C. Slides were washed with PBS at 37 °C and prehybridised with Hybridisation mix. Hybridisation with probes (100 nM) was performed at 54 °C for 18–20 h. Slides were washed twice with 2xSCC for 10 min at 54 °C, followed by a wash with 1x SCC for 10 min at 54 °C and twice washed with 0.2xSCC for 20 min at 37 °C. Slides were blocked with RNAseA at 37 °C. Slides were finally washed with 0.2xSSC for 15 min RT and counterstained with DAPI (1:5000 in PBS). Coverslips were mounted in FluoromountG (Cell Lab, Beckman Coulter). LNA Probes (EXIQON) are listed in Table S6.

Murine Vps39 has two isoforms (NM_147153 and NM_178851), which differ for 1 exon. The Vps39 LNA probe was generated in common region.

RNAseA: 4 ul 10 mg/ml RNAseA, 2 ul 0.5 M EDTA, 100 ul 20XSSC adjusted to 1 ml with DEPC H_2_O.

### Bioinformatic analysis

PUMA (Propagating Uncertainty in Microarray Analysis) package^60, 66–68^ was used for microarray analysis using default settings. PUMA is based on a Bayesian Hierarchical model that accounts for measurements uncertainty and multifactorial design. In this test the error due to multiple testing is controlled through the priors and hence this control is embedded in the overall procedure.

Netview and cytoscape web-based platforms were used to analyse the putative targets. Netview (netview.tigem.it) collects co-expression information for human and murine transcripts^29^. We queried the Netview network with the 132 mouse probesets (127 unique gene symbols, see Input probe sets in Supplementary Table S3) and obtained a corresponding mouse sub-network displaying 78 nodes (see mouse subnetwork in Supplementary Table S3).

The hierarchical clustering was obtained applying the function hclust (under the R environment, http://www.r-project.org/) to the adjacency matrix, by selecting binary (jaccard) as distance between genes.

Cytoscape^30^ is an open source platform for visualizing molecular interaction networks and integrating with gene expression profiles and other data. Cytoscape visualization was obtained by applying the spring edge-weighted (over mutual information -MI- scores) spring embedded layout (www.cytoscape.org) (Supplementary Table S3).

Database for Annotation, Visualization and Integrated Discovery (DAVID) v6.7 (http://david.abcc.ncifcrf.gov/) was used to perform Gene Ontology enrichment analysis as described^69^.

### RNA binding experiments

The antibody against eIF4E was immobilized with protein A/G –Sepharose resin. A Flag – tag resin (Sigma A2220) was used to immunoprecipitate 3XFLAG-OFD1 and 3XFLAG-Bicc1.

Cellular extracts containing approximately 1 mg of silenced (OFD1 or Bicc1) HEK293 transfected as described in supplemental information were precleared on beads (20 uL) in 200 uL of RBB for 1 h at 4 °C to remove RNAs and proteins that bind the beads in a non-specific fashion. The lysate is then loaded on A/G –Sepharose resin and incubated in the appropriate buffer for Ip and Co-Ip, as described above. Any unbound protein is removed by washing three times with the respective buffers and three times with an RNA binding buffer (RBB)/0.1% NP-40 (RBB buffer: 10 mM Tris-HCl pH 7.5, 1.5 mM MgCl_2_, 150 mM KCl, 2 ug/mL lupeptin, 0.5% (v/v) aprotinin and 0.2 mM PMSF), followed by two washes with RBB.

The immunoprecipitated proteins were then incubated ON with 30 ug of total RNA extracted from HEK293 cells in RBB buffer. The complexes were incubated with RBB/0.1% NP-40 with 1 mg/mL heparin for 10 min at 4 °C (the heparin wash minimizes non-specific RNA-protein interactions). The beads are washed four times in RBB/0.1% NP-40 and bound RNA is eluted from the beads by addition of 200 uL of TES buffer (TES: 10 mM Tris-HCl pH 7.5, 1 mM EDTA, 1% sodium dodecyl sulfate (SDS)) and boiled for 3 min. The RNA is phenol/chloroform (1/1) extracted, with 20 ug of glycogen as a carrier. The amount of RNA bound to proteins is analyzed using Real Time-PCR.

## Electronic supplementary material

Supplementary info

Supplementary Table S1

Supplementary Table S2

Supplementary Table S3

Supplementary Table S4

Supplementary Table S5
