# Supplementary material for: The centrosomal OFD1 protein interacts with the translation machinery and regulates the synthesis of specific targets
Source: Sci Rep. 2017 Apr 27;7:1224. doi: 10.1038/s41598-017-01156-x (PMC5430665; doi:10.1038/s41598-017-01156-x)

The centrosomal OFD1 protein interacts with the translation machinery and regulates the synthesis of specific targets

Daniela Iaconis[1], Maria Monti[2], Mario Renda[1], Arianne van Koppen[3], Roberta Tammaro[1], Marco Chiaravalli[4], Flora Cozzolino[2], Paola Pignata[1], Claudia Crina[1], Piero Pucci[2], Alessandra Boletta[4], Vincenzo Belcastro[1], Rachel H Giles[3], Enrico Maria Surace[1], Simone Gallo[5], Mario Pende[6], Brunella Franco[1,7]

SUPPLEMENTARY INFORMATION

Contains supplemental figures (S1 to S6), legend to Supplemental tables and supplemental table S6 (primers used in the study).

Supplementary Tables

Supplementary Table 1. Sequences and features of the peptides identified in proteomic studies. Sheet 1 displays information on sequences and features of each peptide used for the identification of the putative interacting proteins from the cells expressing 3XFLAG-OFD1. In sheet 2 are illustrated the protein immunoprecipitated in the control experiment. The control experiment obtained by immunoprecipitation of the empty vector transfected cells with anti-FLAG agarose beads allowed ruling out nonspecific retained proteins. Proteins are indicated with the UniProt (www.expasy.org) code number.

Supplementary Table 2. Microarray analysis of polysomal RNAs. The Table shows the targets differentially expressed in polysomal mRNAs extracted from kidneys of *Ofd1*-IND compared to Controls at P8. The underrepresented targets are highlighted in green, while the

enriched RNAs in red.

Supplementary Table 3. Input probesets used for the bioinformatic analysis of targets are listed. For each probeset the Official Gene name is indicated.

Supplementary Table 4. The mouse subnetwork and the MI scores are reported.

Supplementary Table 5. Gene Clusters . The genes belonging to the two clusters are reported in the table with information from microarray analysis. Cluster 1 is highlighted in yellow, while Cluster 2 in blue.

Supplementary Table 6. Primers

**PRIMERS for Mutagenesis**	
OFD1_4EBS_YS Fw	GCACCAAGAGATTGAAACAAAAGAAATTTCTGCTCAAAGGCAAC
OFD1_4EBS_YS Rw	GTTGCCTTTGAGCAGAAATTTCTTTTGTTTCAATCTCTTGGTGC
OFD1_4EBS_YD Fw	GCA CCAAGAGATTGAAACAAAAGAAATTGATGCTCAAAGGCAAC
OFD1_4EBS_YD Rw	G TTGCCTTTGAGCATCAATTTCTTTTGTTTCAATCTCTTGGTGC
**PRIMERS for Real-Time and RT PCR**	
OFD1 Fw	TCTTTCCAGAAAGTGGTTTGG
OFD1 Rv	GAGACTGGAAGTAGGG TTGATTTT
FireflyLuciferase Fw	TATCGGAGTTGCAGTTGCGC
FireflyLuciferase Rv	TCGACTGAAATCCCTGGTAATC
Renilla Fw	TGCCACATATTGAGCCAGTAG
Renilla Rv	CCAATCATGGCCGACAAAAATG
Gh Fw	AATGCCCAGGCTGCTTTCTG
Gh Rv	TGTTGGTGAAAATCCTGCTGAG
Vps39 Fw	TTGAGGTAACACTAGAGAAGTCC

Vps39 Rv	TGGAGGTCACAGGTGAACAG
Gapdh Fw	TCTTCTGGGTGGCAGTGAT
Gapdh Rv	TGCACCACCAACTGCTTAGC
GAPDH Fw	ATGTTCGTCATGGGTGTGAA
GAPDH Rw	AGGGGTGCTAAGCAGTTGGT
Net1 Fw	AGCTGAAGAATAAAAGCGGCC
Net1 Rv	TTCCAGCACCAGCTCCTG
Gdi2 Fw	TCAGTTGGTGAAGATGCTGC
Gdi2 Rv	TCTGCTTCAGTGGAAGGAAC
Vcl Fw	TGATTGATGAGAGACAGCAGG
Vcl Rv	TGAAATGAGAACAGGAAGCAGC
**LNA Probes are from EXIQON**	
Controls probe scrambled-ISH	56-FAM/GTGTAACACGTCTATACGCCCA
Exon-1-*Net1*	56-FAM/ACCGATCGTGGCGGGCACAA
Exon-1-*Gdi2*	56-FAM/ATCACGTCGTATTCCTCATTCA
Exon-2-*Vps39* (antisense)	56-FAM/AACATCCTTCCGAATCCTGTAGA
Exon-2-*Vps39* (sense)	56-FAM/TCTACAGGATTCGGAAGGATGTT
Exon-2-*Gh*	56-FAM/AGGGCATGGCGGGAAAAGCACT

Supplementary Figures

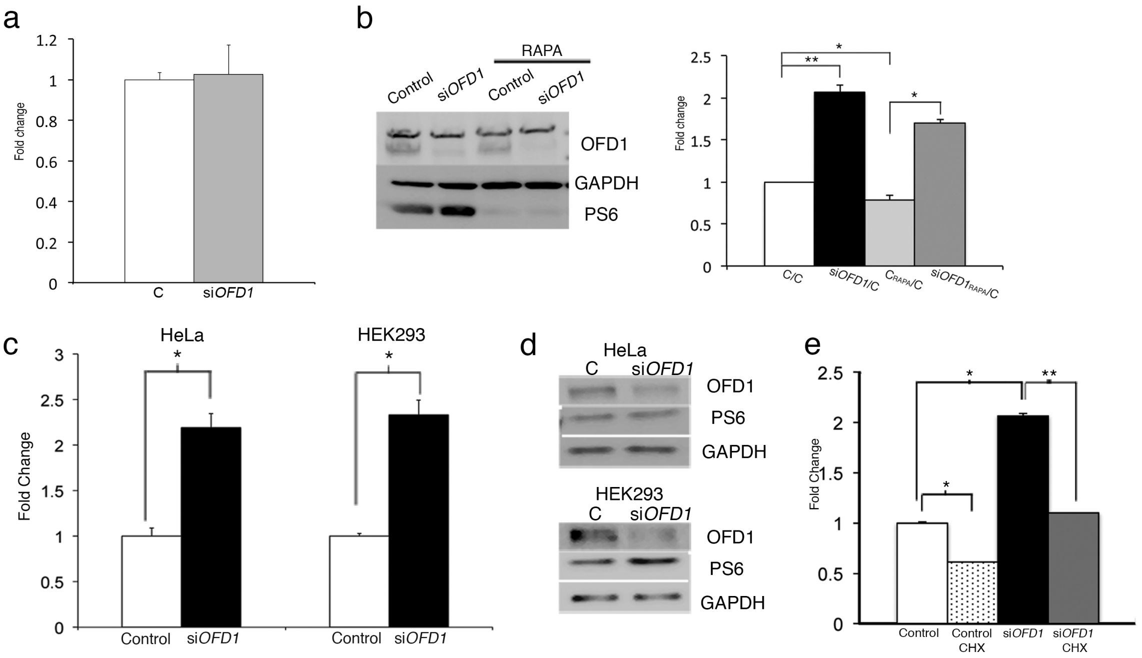

Supplementary Fig.1. Cap-dependent translation is upregulated in *OFD1*-silenced HEK293 cells. (a) Real-Time PCR experiment revealing levels of luciferase expression in control (white bar) and *OFD1*-silenced cells (grey bar) used for experiments displayed in Fig 2a. (b) (Left) Control and *OFD1*-silenced HEK293 cells were transfected with the bicistronic reporter plasmid pRL-HCV-FL and treated with rapamycin (RAPA). WB was used to evaluate the effect of rapamycin on the Phosphorylation of the rpS6 protein (PS6). (Right) RAPA treatment decreased Renilla accumulation both in control and *OFD1*-silenced cells (c) Control and *OFD1*- silenced HeLa (left) and HEK293 (right) cells were transfected with the pRL-HCV-FL construct. In both systems Renilla/firefly luciferase light-unit ratio was calculated; the value of control cells (Control; white bar) was set at 1 and the fold change for *OFD1*- silenced cells (si*OFD1*; black bar) was calculated and reported in the graph. (d) WB analysis showed that *OFD1*-silenced HeLa cells (top panel) do not show the increase in rpS6 phosphorylation (PS6) observed in

HEK293 cells (lower panel). (e) Both control and *OFD1*-silenced HEK293 cells were treated with cyclohexymide (CHX) for 5 hours. The rate of degradation is comparable between Control (dotted bar) and *OFD1*-silenced cells (grey bar). Data are presented as the mean ± SEM. *p-value<0.05; **p-value<0.01. For all assays *n*=3.

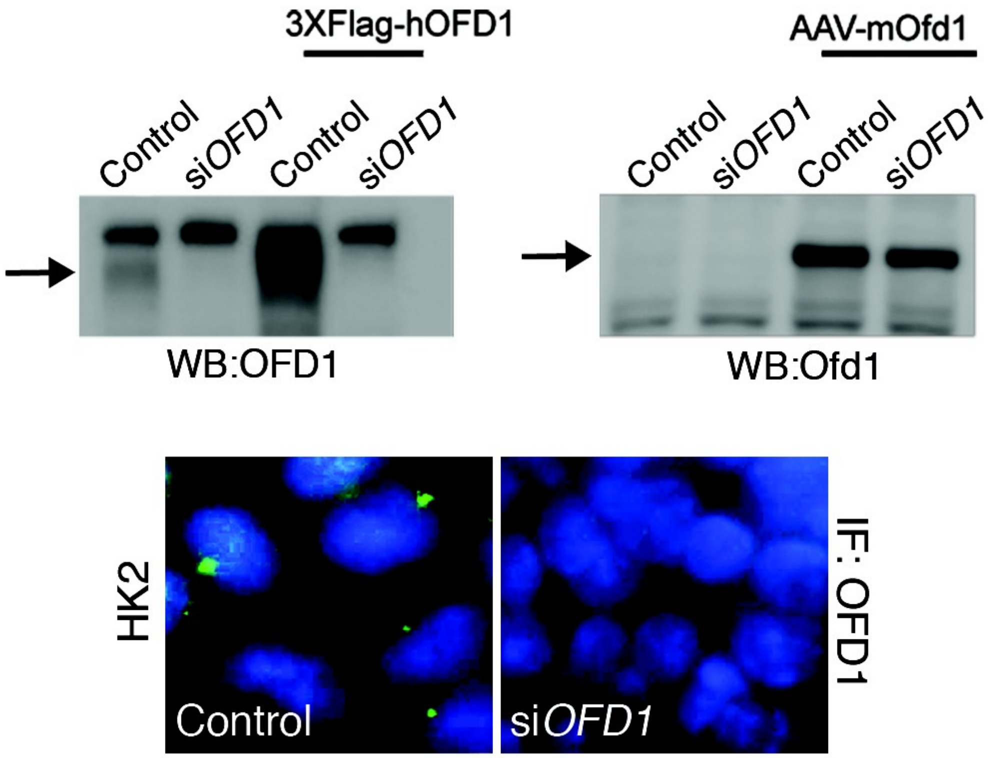

Supplementary Fig.2. Controls for OFD1 siRNA and antibody specificity. (Upper panels) Left panel. The OFD1 protein levels increase in cells overexpressing the human *OFD1* transcript (3XFLAG-hOFD1) and almost disappear in *OFD1*-siRNA treated cells. Right panel, WB analysis using an antibody, which specifically recognises the murine Ofd1, reveals that OFD1-silenced and control HEK293 cells in which the murine Ofd1 was overexpressed (AAV-m*Ofd1*) display increased amount of murine Ofd1 protein. No murine protein could be detected in untransfected HEK293 *OFD1*-silenced and control cells. This experiment demonstrates that the murine *Ofd1* transcript is insensitive to the siRNA targeting the human transcript. Bottom panels. The specificity of the antibody against the human OFD1 was evaluated by IF in HK2 cells. The experiment revealed that the OFD1 signal disappeared in siRNA treated cells.

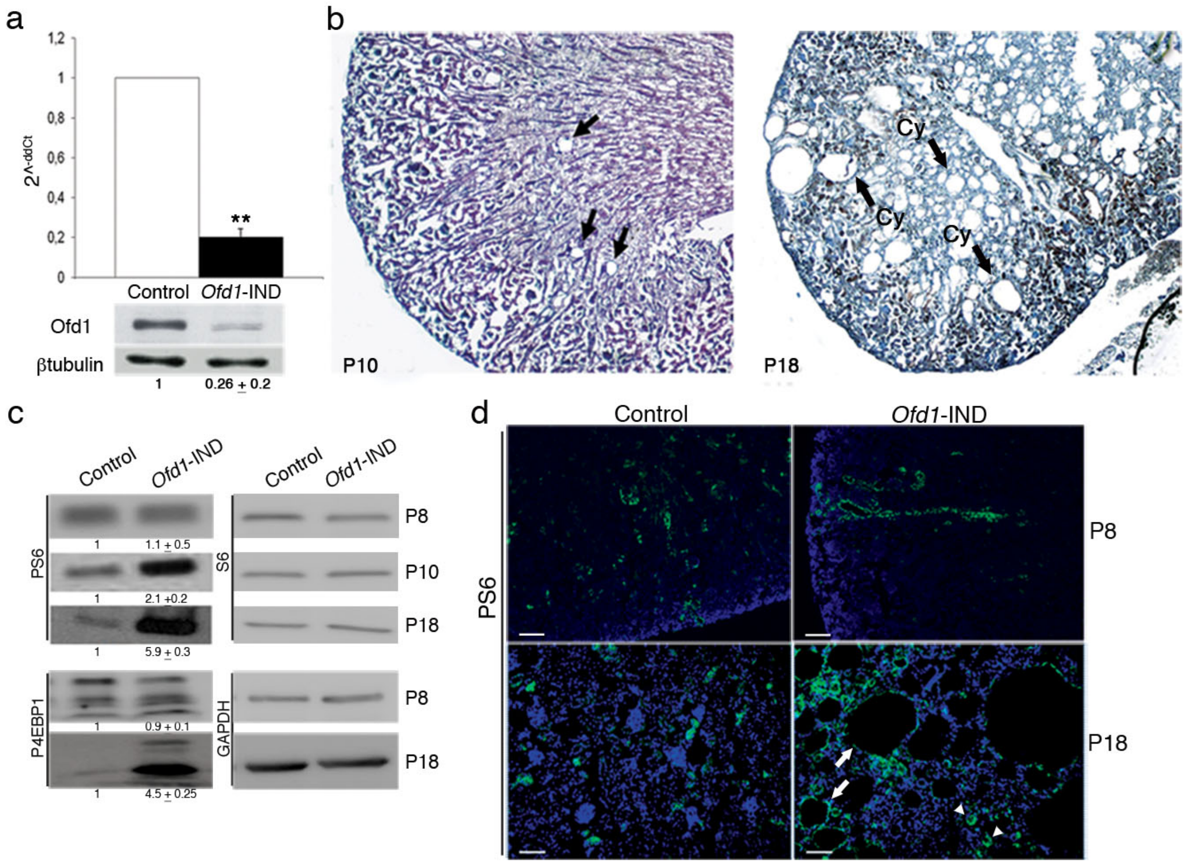

Supplementary Fig.3. Characterization of the Ofd1fl/y;CAGGcre$^{ER\text{-}TM}$ (*Ofd1*-IND) mice. (a) *Ofd1* inactivation in the *Ofd1*-IND model. Real-Time PCR and WB with an antibody against the murine Ofd1 protein on kidney lysates from controls and Ofd1$^{fl/y}$;CAGGcre$^{ER\text{-}TM}$ (*Ofd1*-IND) mice at P18 indicate 80% inactivation of the *Ofd1* transcript. (b) Cyst progression in the *Ofd1*-IND model. Hematoxylin-eosin staining of kidney sections from *Ofd1*-IND mutants at different stages. At P10, dilated tubules appear (arrows). Cysts (Cy and black arrows) rapidly increase in size and number, replacing the majority of the parenchyma at P18. (c) Analysis of the mTORC1 pathway in the *Ofd1*-IND model on kidney lysates. WB experiments on renal lysates at different stages show higher amounts of phosphorylated rpS6 (PS6) in *Ofd1*-IND mice, which is evident from P10. Analysis of Phospho4EBP1 (P4EBP1), another readout

of the mTORC1 pathway activation, shows an evident accumulation in kidneys from *Ofd1*-IND mice at P18 while at P8 the levels of P4EBP1 where comparable between controls and mutants. Total rpS6 (S6) protein and GAPDH were used to normalize the results. The fold change was calculated and presented as the mean ± SEM below the panels. (d) Analysis of the mTORC1 pathway in the *Ofd1*-IND model on kidney sections. Immunofluorescence analysis of PS6 on kidney sections from Control and *Ofd1*-IND mice at P18 reveals increased amounts of PS6 in cells lining a number of cysts and non-dilated tubules (white arrows). Bar=50μm. **p-value<0.05.

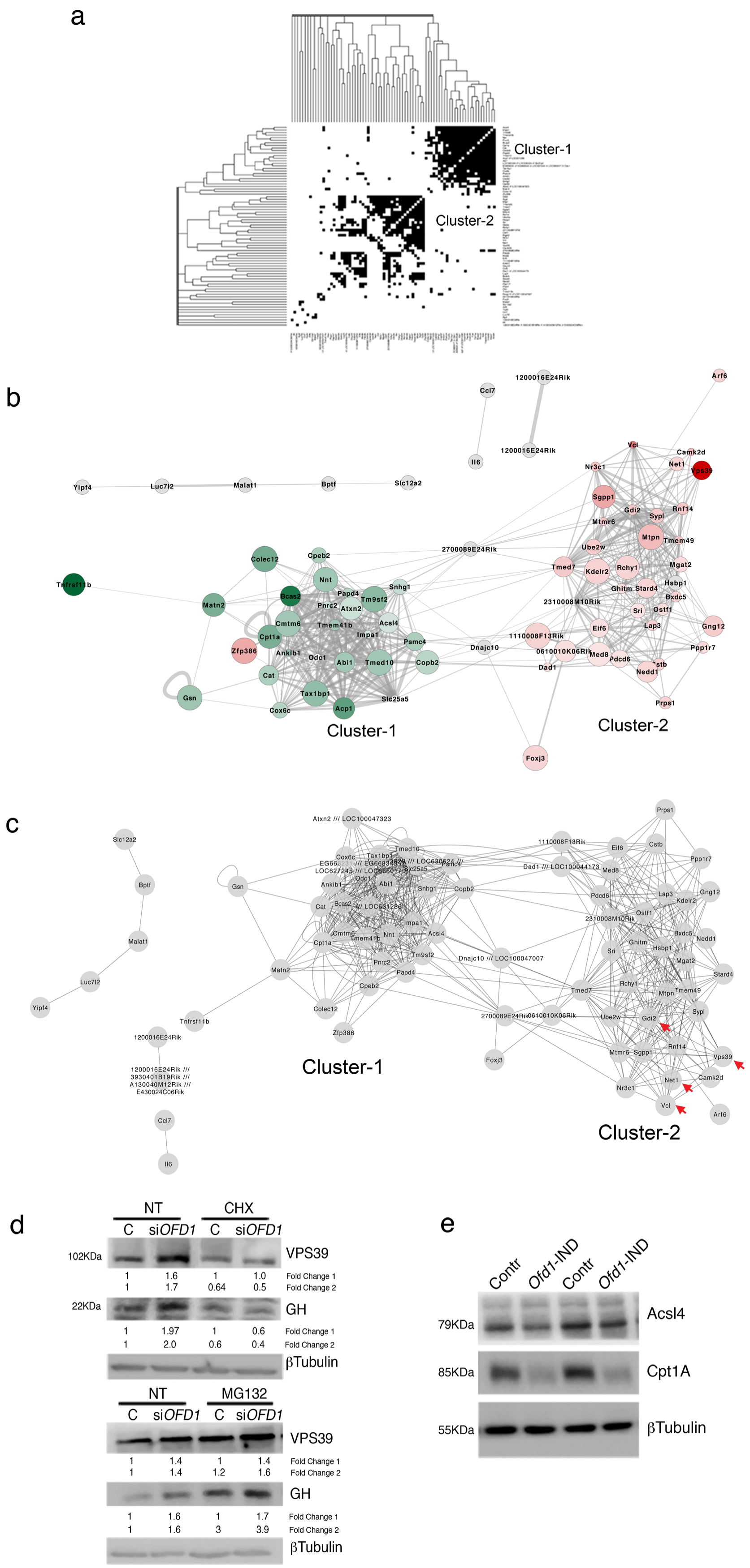

a
Cluster-1
Cluster-2
b
Cluster-1
Cluster-2
c
Cluster-1
Cluster-2
d
NT
CHX
C
siOFD1
VPS39
102KDa
Fold Change 1
Fold Change 2
22KDa
GH
βTubulin
MG132
e
Contr
Ofd1-IND
79KDa
Acsl4
85KDa
Cpt1A
55KDa
βTubulin

Supplementary Fig.4. Analysis of OFD1 translational targets. (a) Hierarchical clustering of the mouse sub-network adjacency matrix (see Table S3 and S4). Two major clusters, named Cluster-1 and Cluster-2, can be appreciated. (b) Cytoscape visualization of the mouse sub-network obtained from microarray data (see also Supplementary Table 2 and Table 4). The network was arranged automatically with the “organic layout” built-in Cytoscape function. The Cytoscape visualisation shows the same two major clusters. Node colors were chosen to reflect the log2 of the Fold Change (FC) from microarray analysis (green and red correspond to negative and positive FC, respectively). The intensity of the colours reflects the FC: darker colours correspond to higher FC values. The size of the nodes reflects the significance: bigger nodes are associated with a lower p value. The grey colour indicates nodes outside the clusters. (c) The analysis was performed also on the total of 141 targets identified. The same clusters were observed. Targets selected for further validation are indicated by red arrows. Information on cluster content is included in Supplementary Table S5. (d) Control and *OFD1*-silenced cells were treated with CHX, an inhibitor of protein translation (left) and with MG132, an inhibitor of protein degradation (right). The accumulation of proteins observed in *OFD1*-silenced cells was rescued only by CHX treatment (Fold change 1). Protein levels observed in *OFD1*-silenced (treated or not treated) cells were normalized against the respective control (Fold change 1). All samples were normalized against the untreated control (Fold change 2). (e) WB analysis showed the downregulation of Cpt1A and ACSL4 in renal lysates from *Ofd*-IND compared to controls. CHX, cyclohexamide; NT, untreated; C, control.

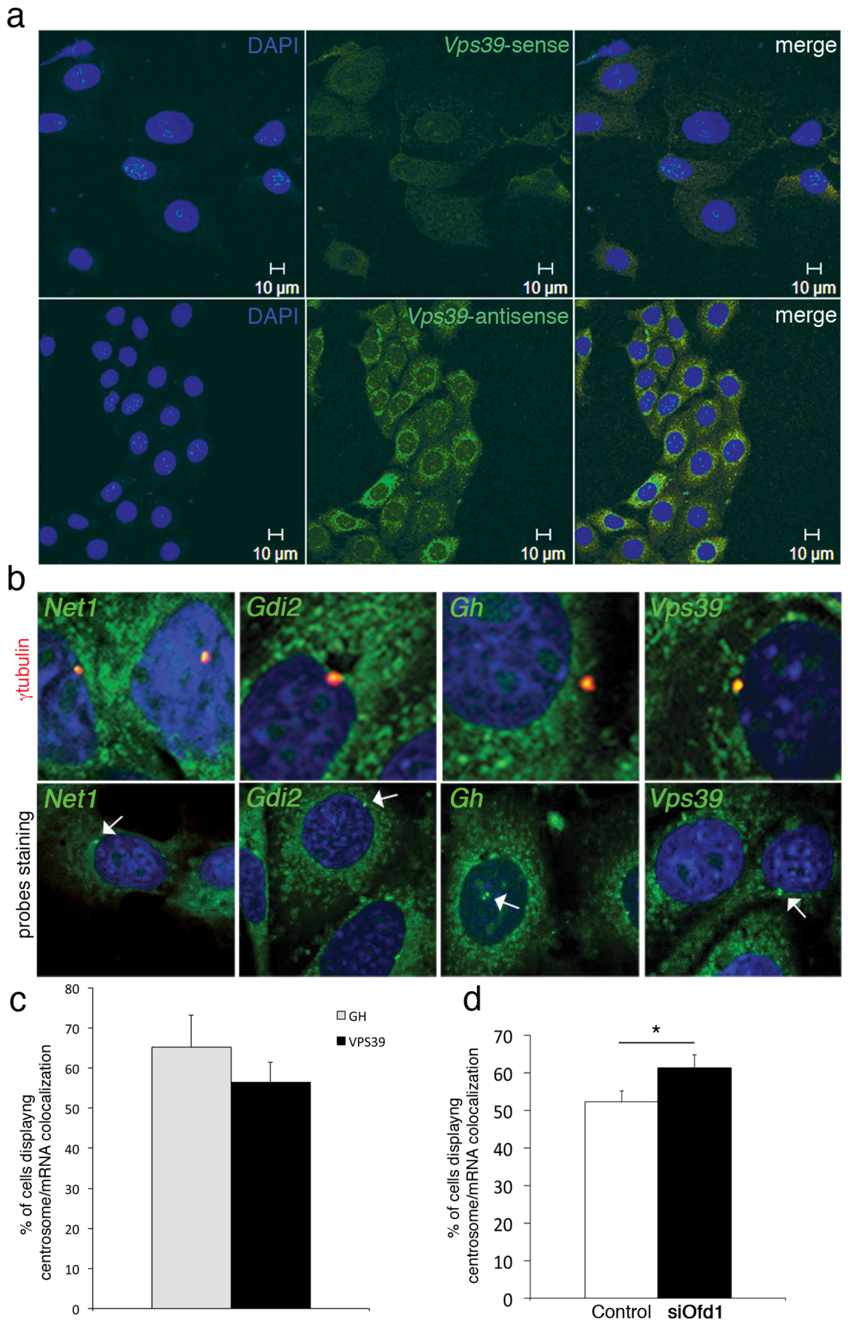

Supplementary Fig.5. Controls for RNA FISH studies. (a) *Vps39* sense and antisense probes (green) were used for ISH experiments and demonstrate the specificity of the signal. (b) Upper panels. Magnification of ISH experiments showing colocalization of Net1, Gd12, Gh, and Vps39 with centrosomal γtubulin marker. Lower panels. Single channel images are presented for each ISH probe analysed, arrowheads indicate the staining that suggest the centrosomal localization. DAPI was used to stain nuclei (blue). Bar=10μm. Representative images were taken at the same contrast. *n*=3. (c) Counting of

the number of cells showing γtubulin/*Gh* (grey bar) and γtubulin/*Vps39* (black bar) mRNA colocalization. (d) Counting of the number of cells showing γtubulin/*Vps39* mRNA colocalization in control cells (white bar) and in *Ofd1*-silenced cells (black bar). *p-value<0.05

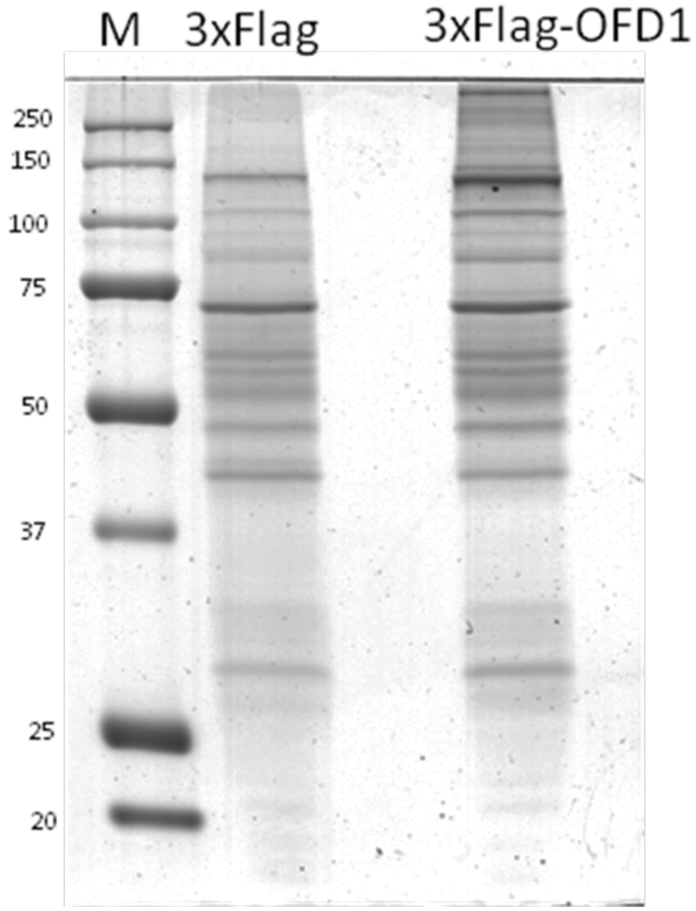

Supplementary Fig.6. Comassie staining. Scanned picture of a Coomassie stained gel from a one-dimensional gel separation of total lysates from HEK293 cells transfected with the 3XFLAG empty vector (left) and the 3xFlag-OFD1 construct (right). M indicates the lane displaying the protein molecular weight standard, the estimated molecular weights are indicated on the left of the protein ladder.

Accession number for the microarray experiment

Username: Reviewer_E-MTAB-2827
Password: QUwgjtgx

Full unedited gel for figure 1B

The red arrows Indicate the bands reported in Figure 1 C

First panel
Lanes 1,2 and 3

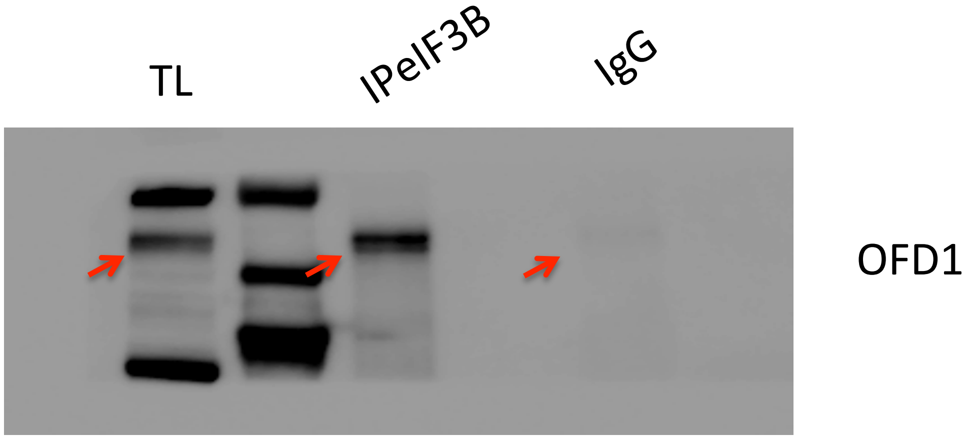

Second panel
Lanes 1,2 and 3

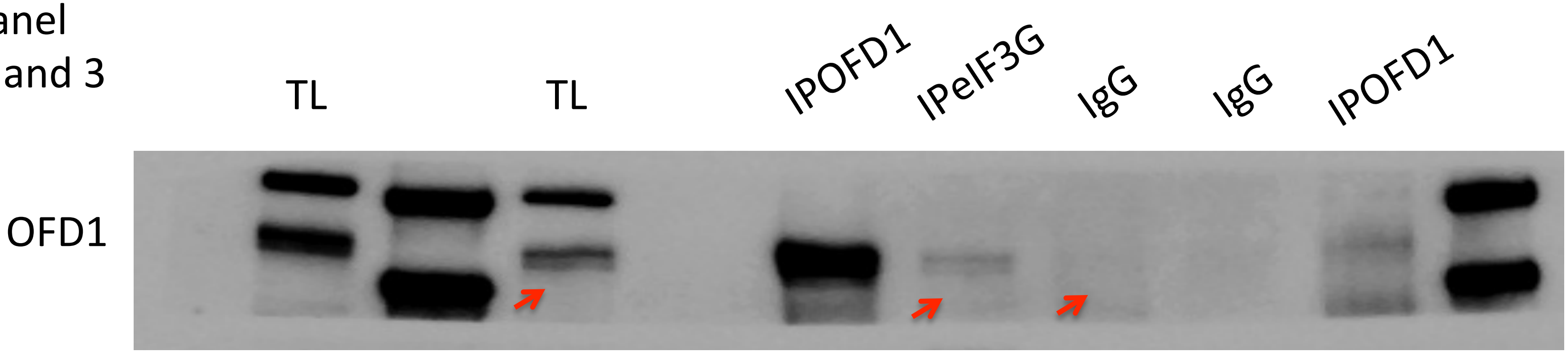

Third panel
Lanes 1,2 and 3

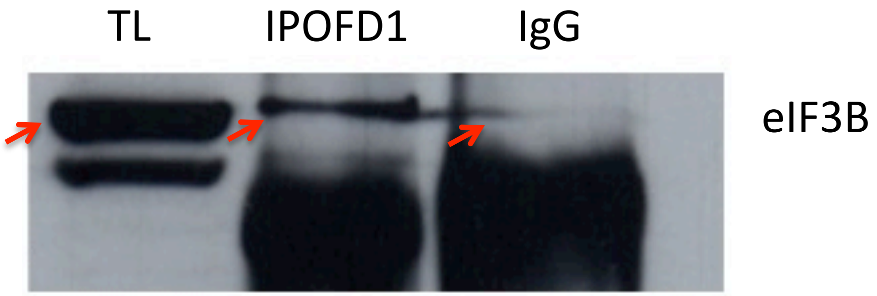

Fourth panel
Lanes 1,2 and 3

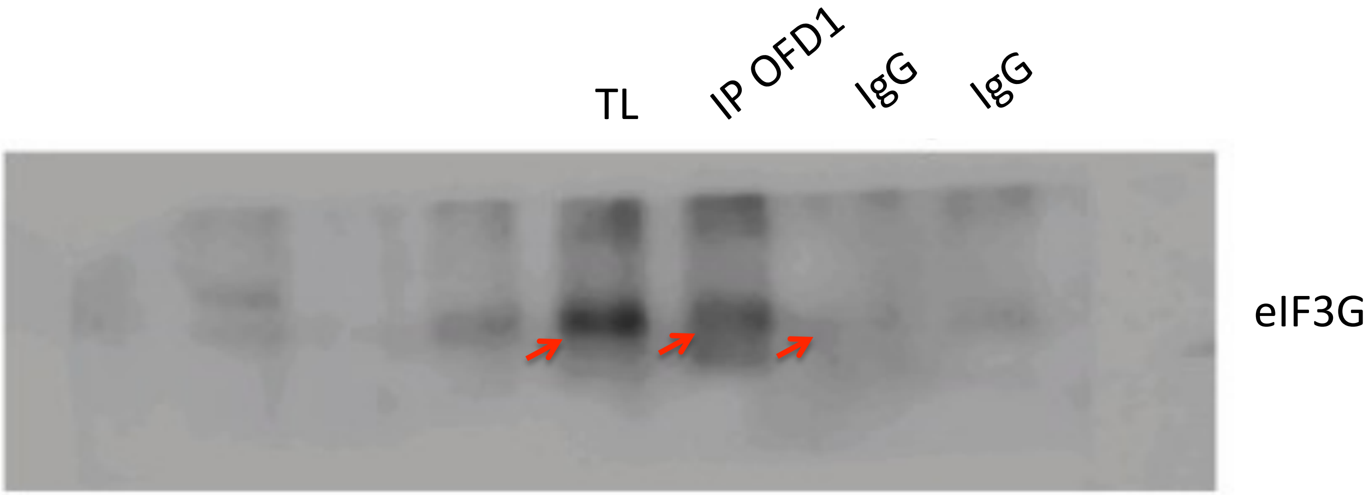

The blue arrows Indicate the IgG bands (not reported in the figures)

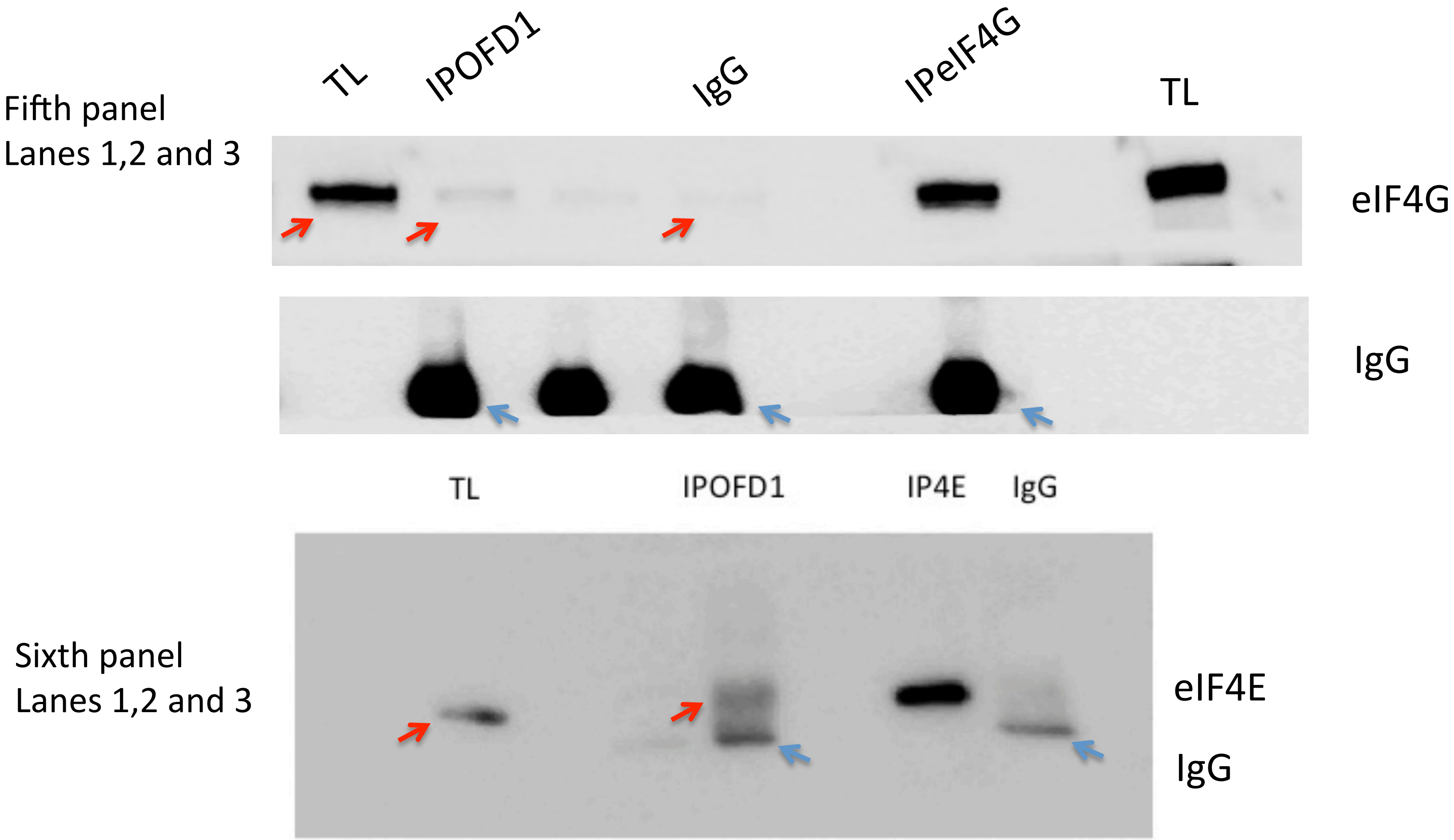

Seventh panel
Lanes 1,2 and 3

LT IPOfd1 IPeIF3n IgG

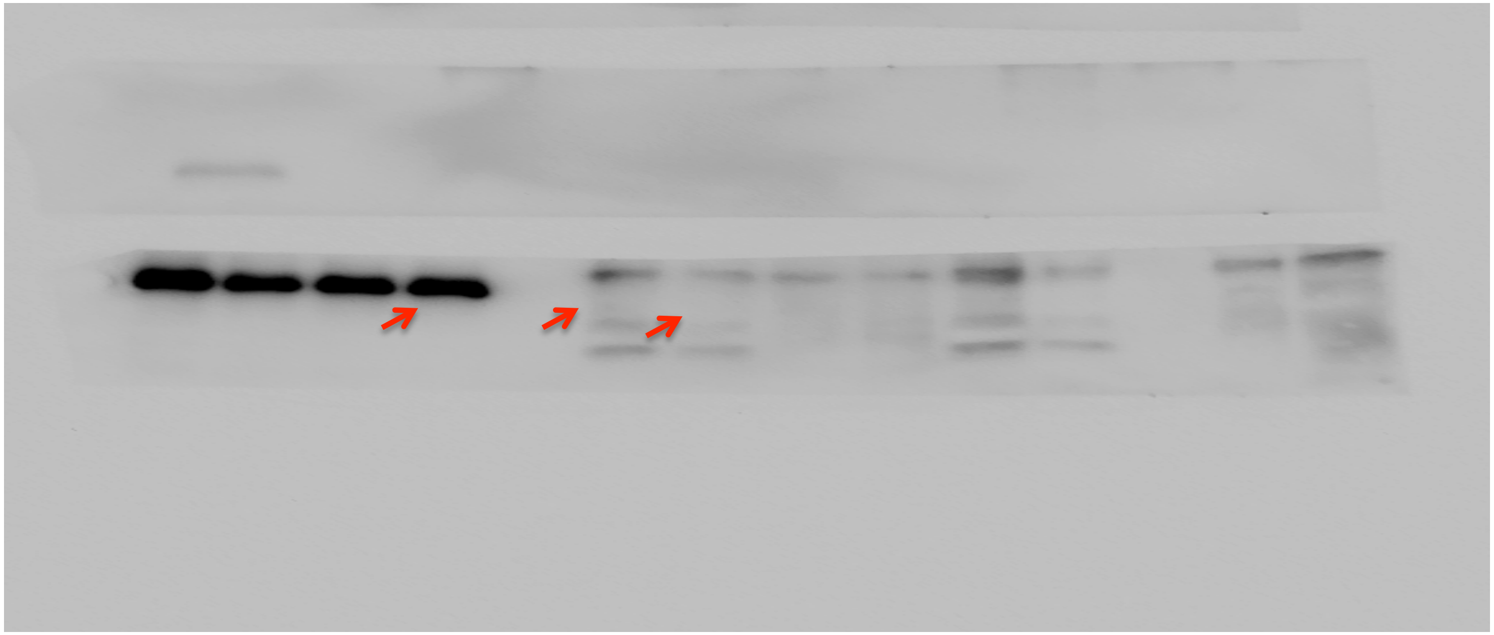

HCCS

Eighth panel
Lanes 1,2 and 3

LT IPOfd1 IPeIF3n IgG

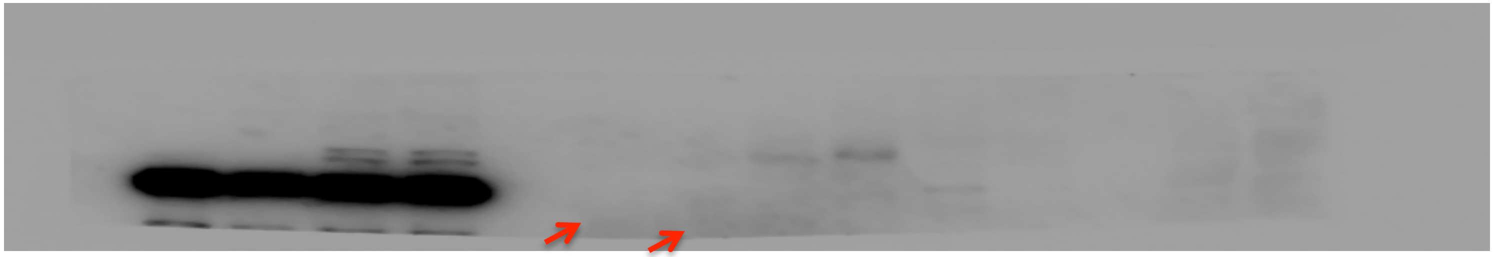

CS

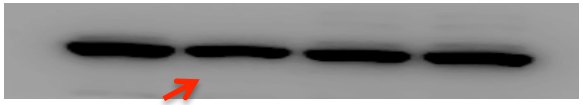

Shorter exposure

Full unedited gel for figure 1C

The red arrows Indicate the bands reported in Figure 1 C

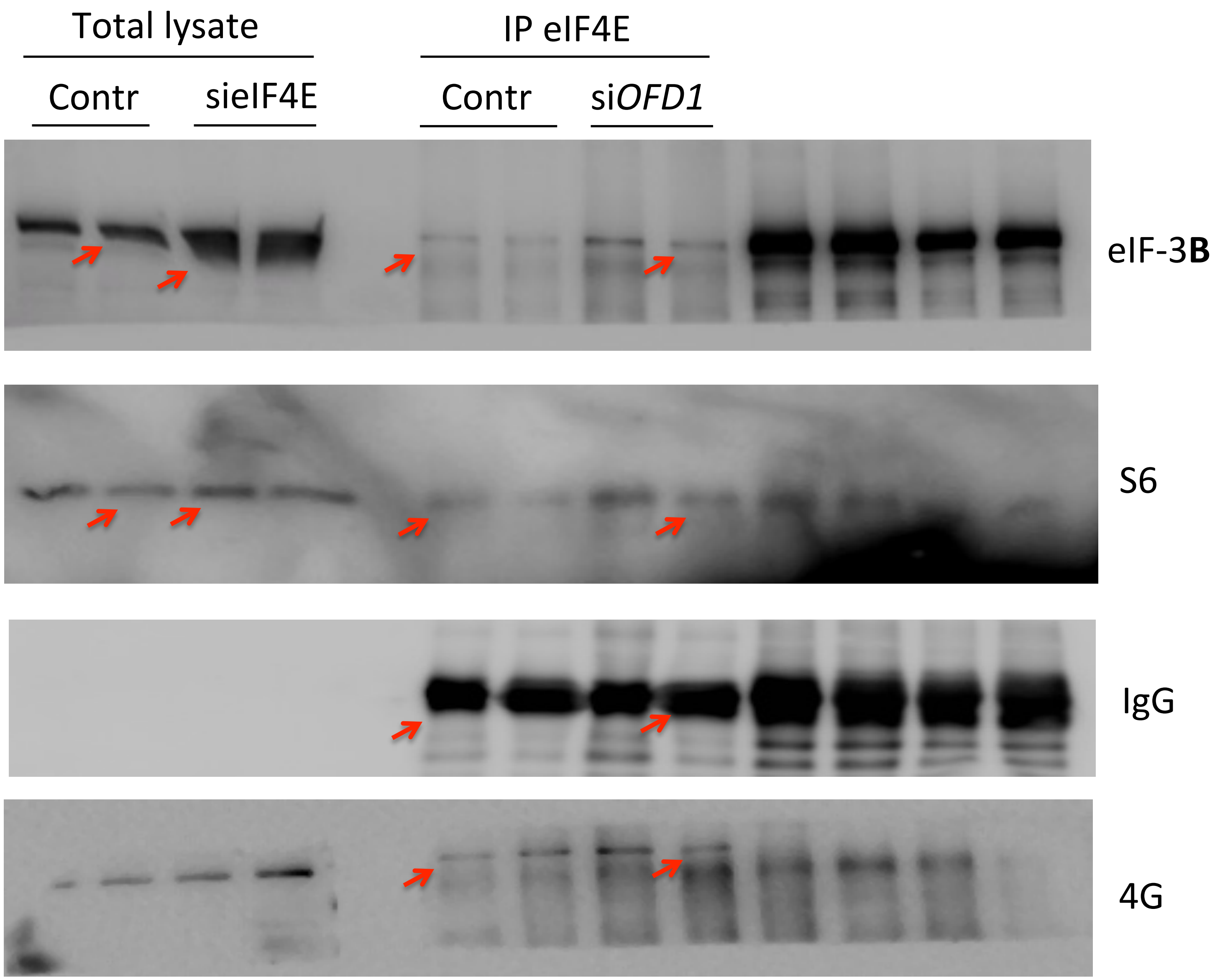

Full unedited gel for figure 1D

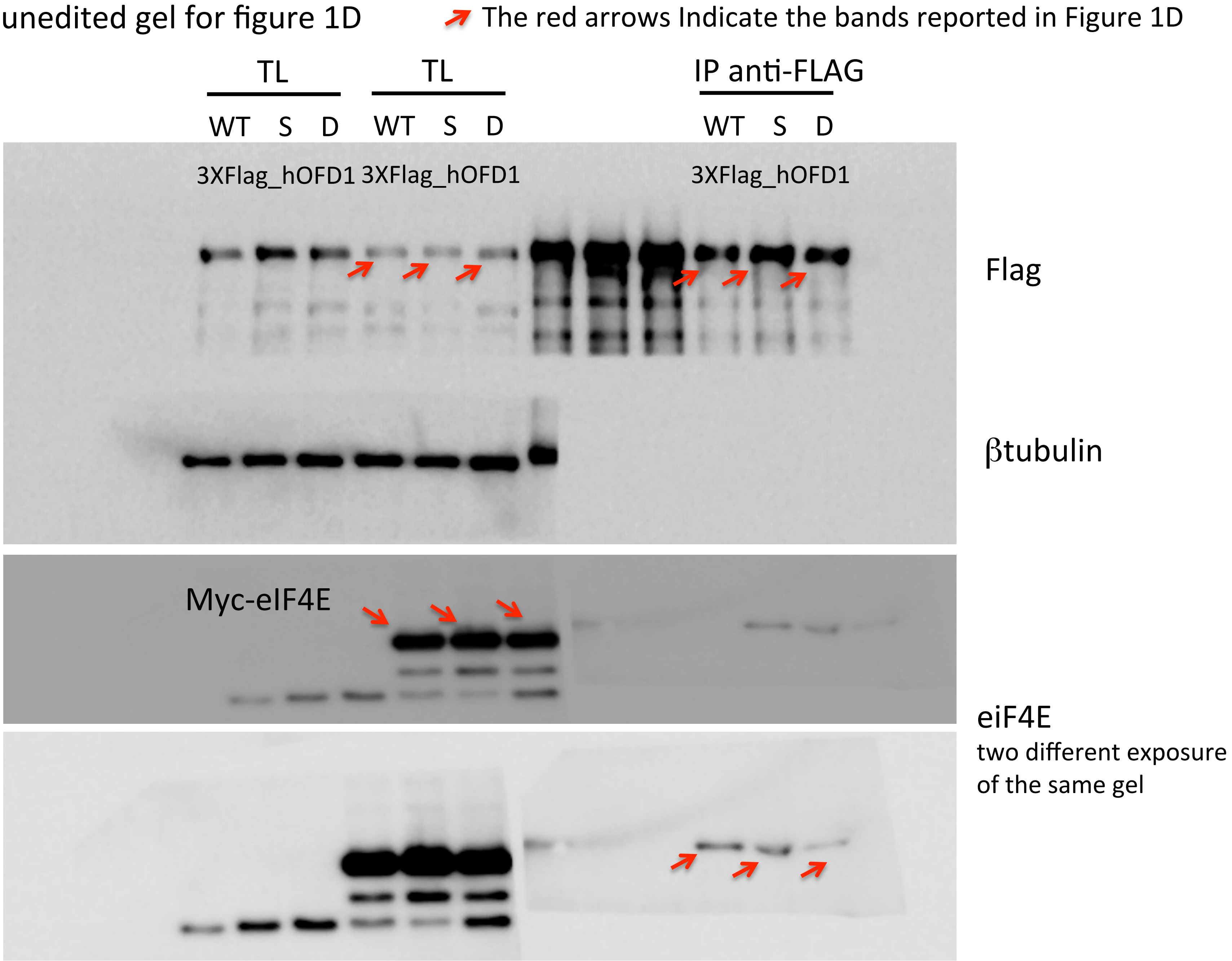

Full unedited gel for figure 1E

The red arrows Indicate the bands reported in Figure 1E

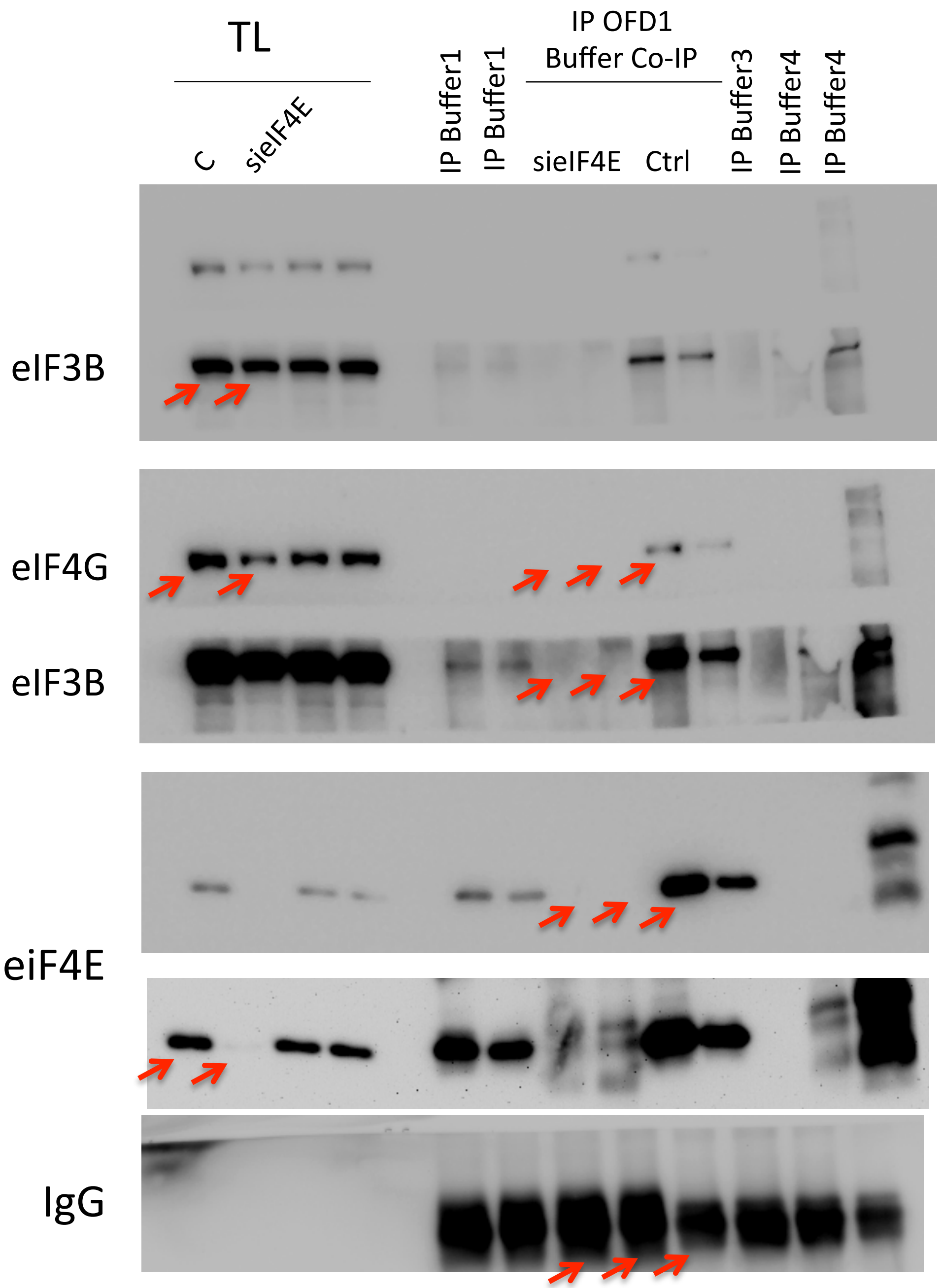

two different exposure of the same gel

two different exposure of the same gel

Full unedited gel for figure 3A

The red arrows Indicate the bands reported in Figure 3A

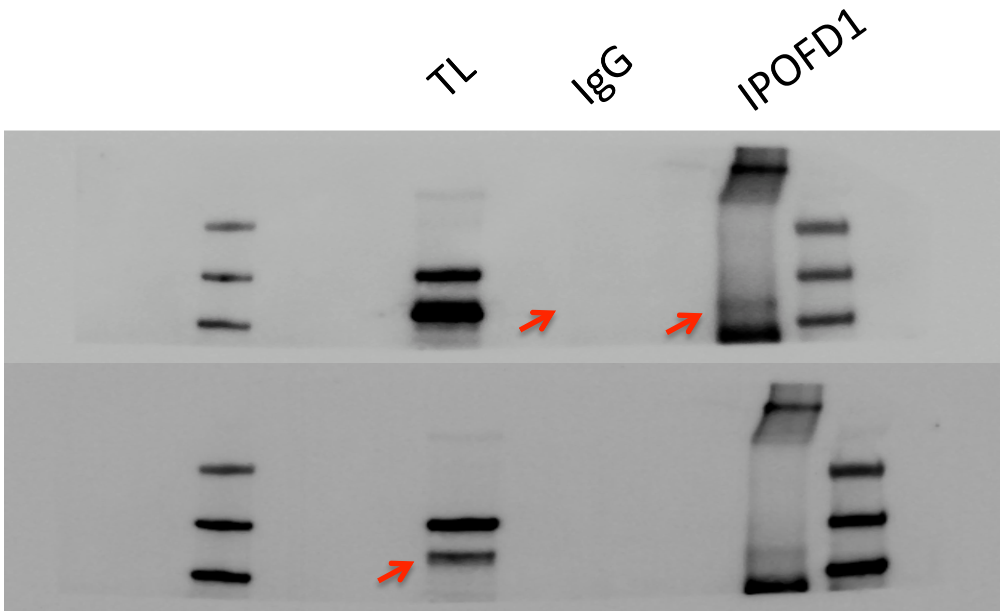

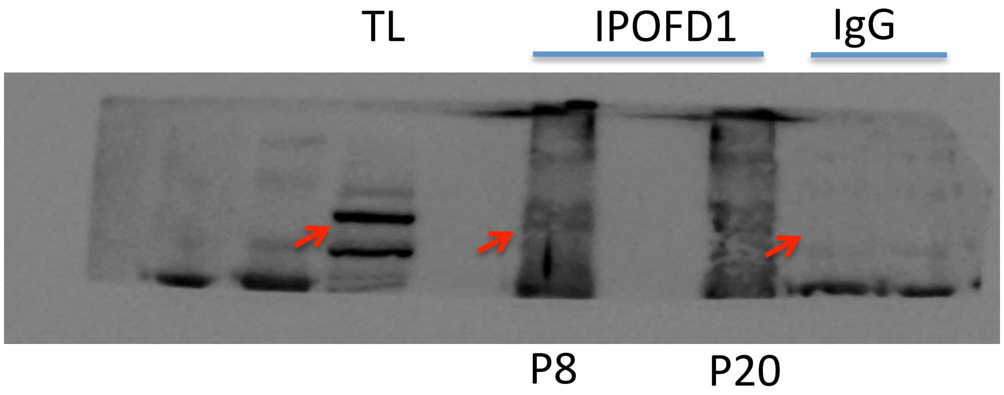

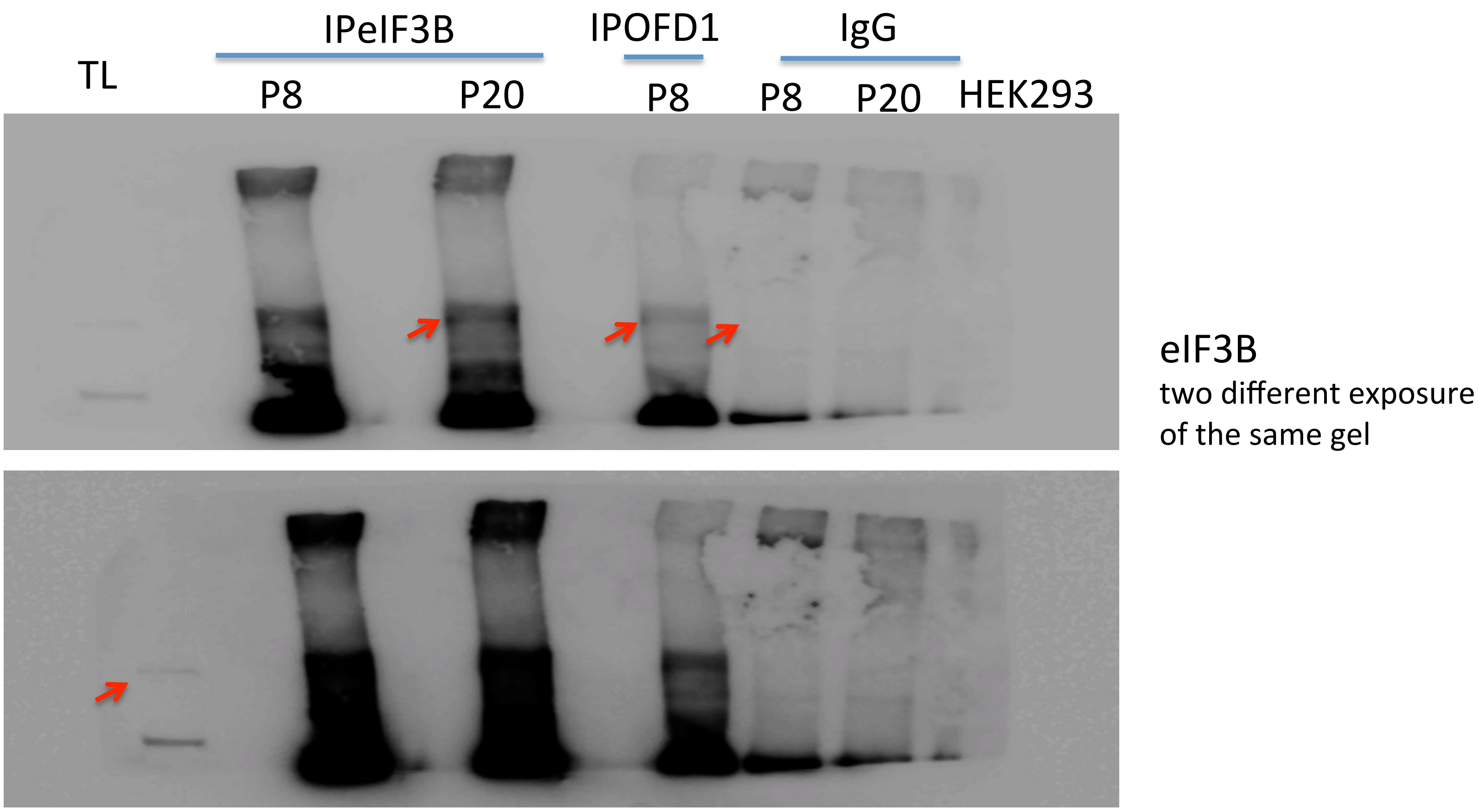

Third panel
Lanes 1,2,3 and 4

Fourth panel
Lanes 1,2,3 and 4

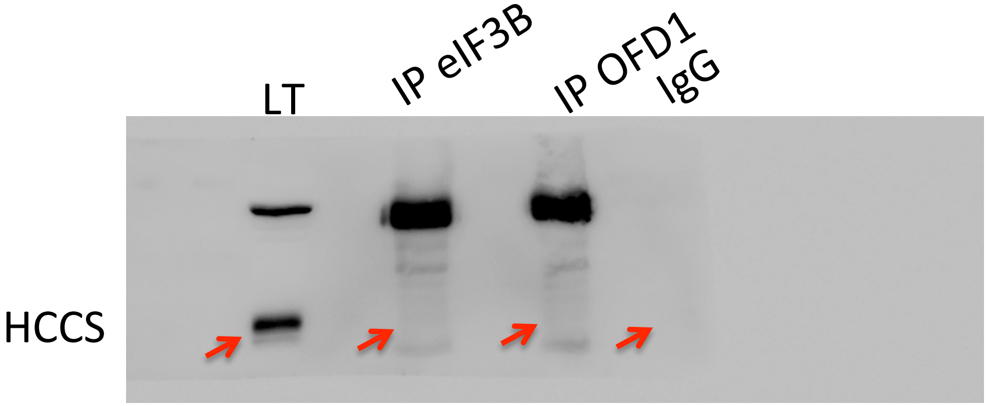

Fifth and sixth panels
two different exposure
of the same gel

Lanes 2 and 1

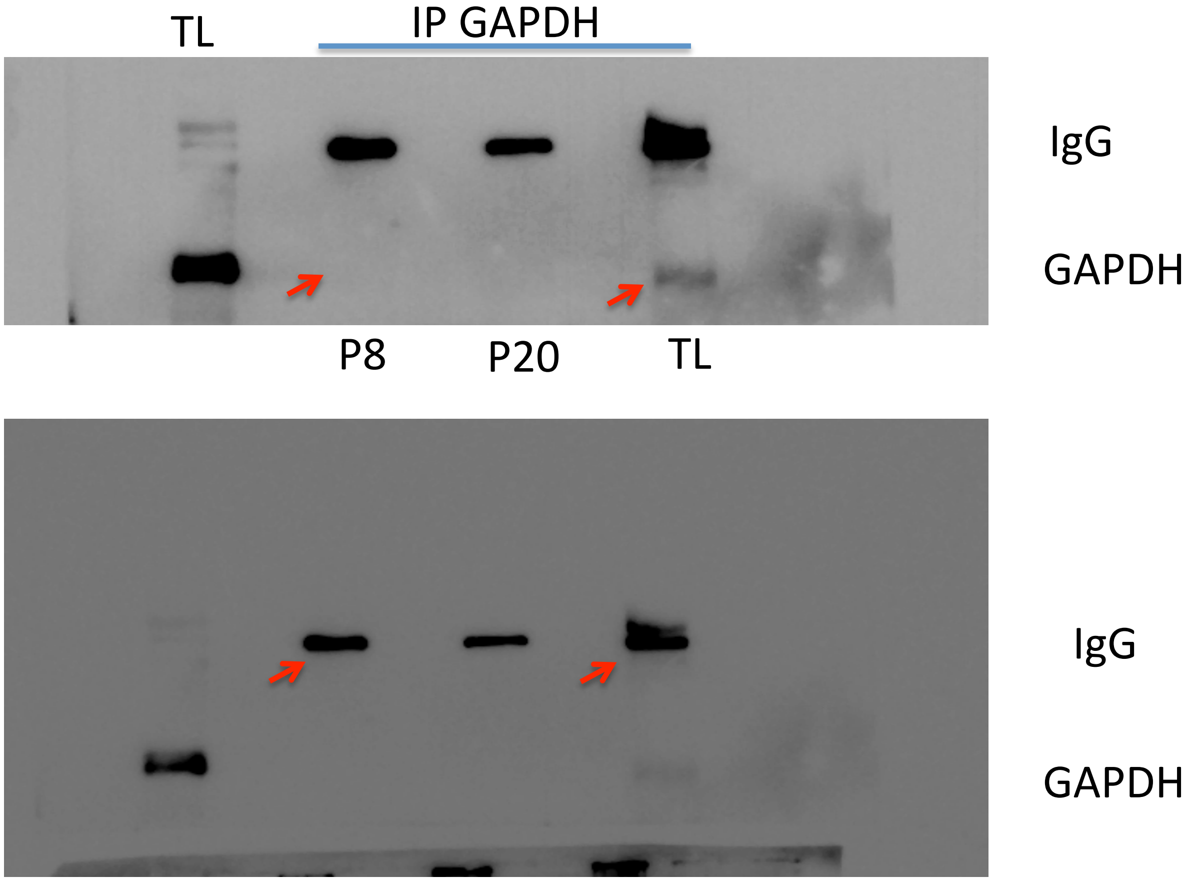

Seventh and eighth panels

Lanes 1 and 2

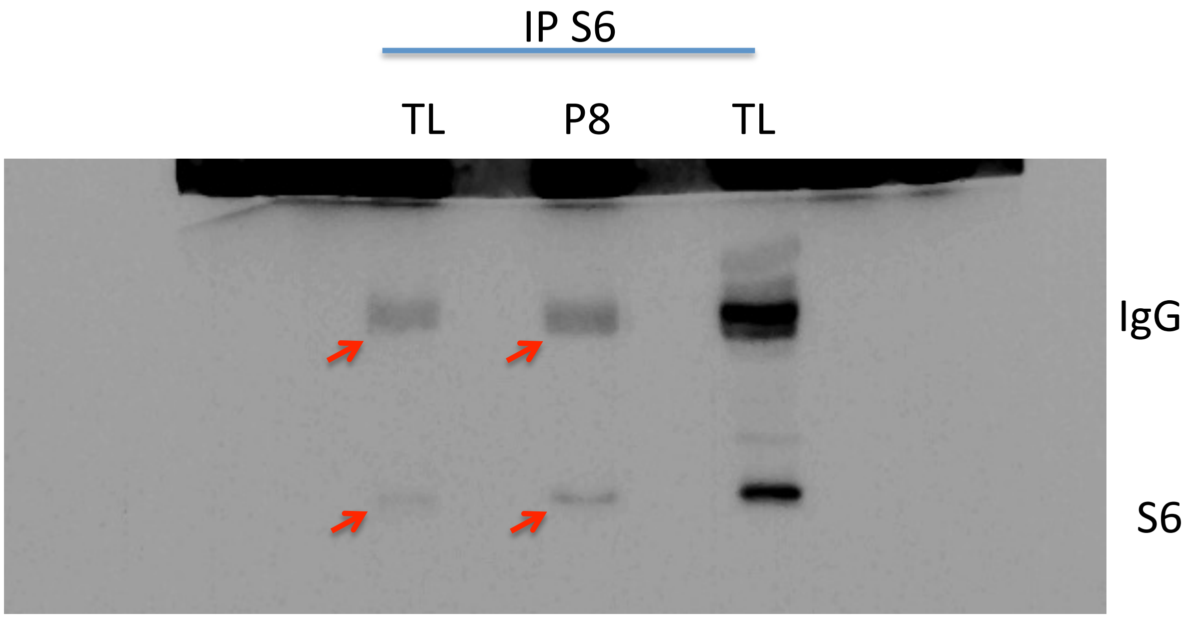

Full unedited gel for figure 4C

The red arrows Indicate the bands reported in Figure 4C

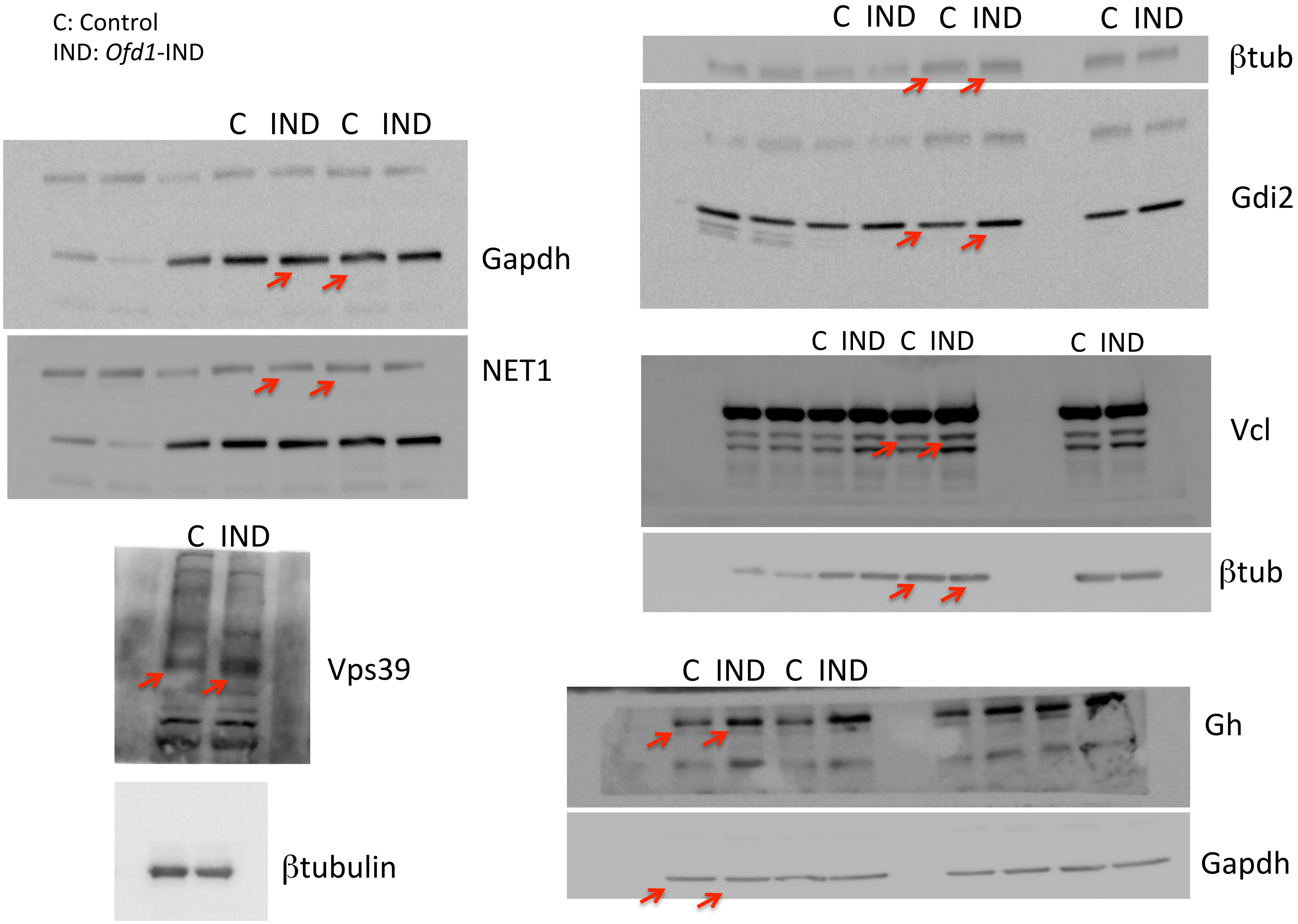

Full unedited gel for figure 4E

The red arrows Indicate the bands reported in Figure 4E

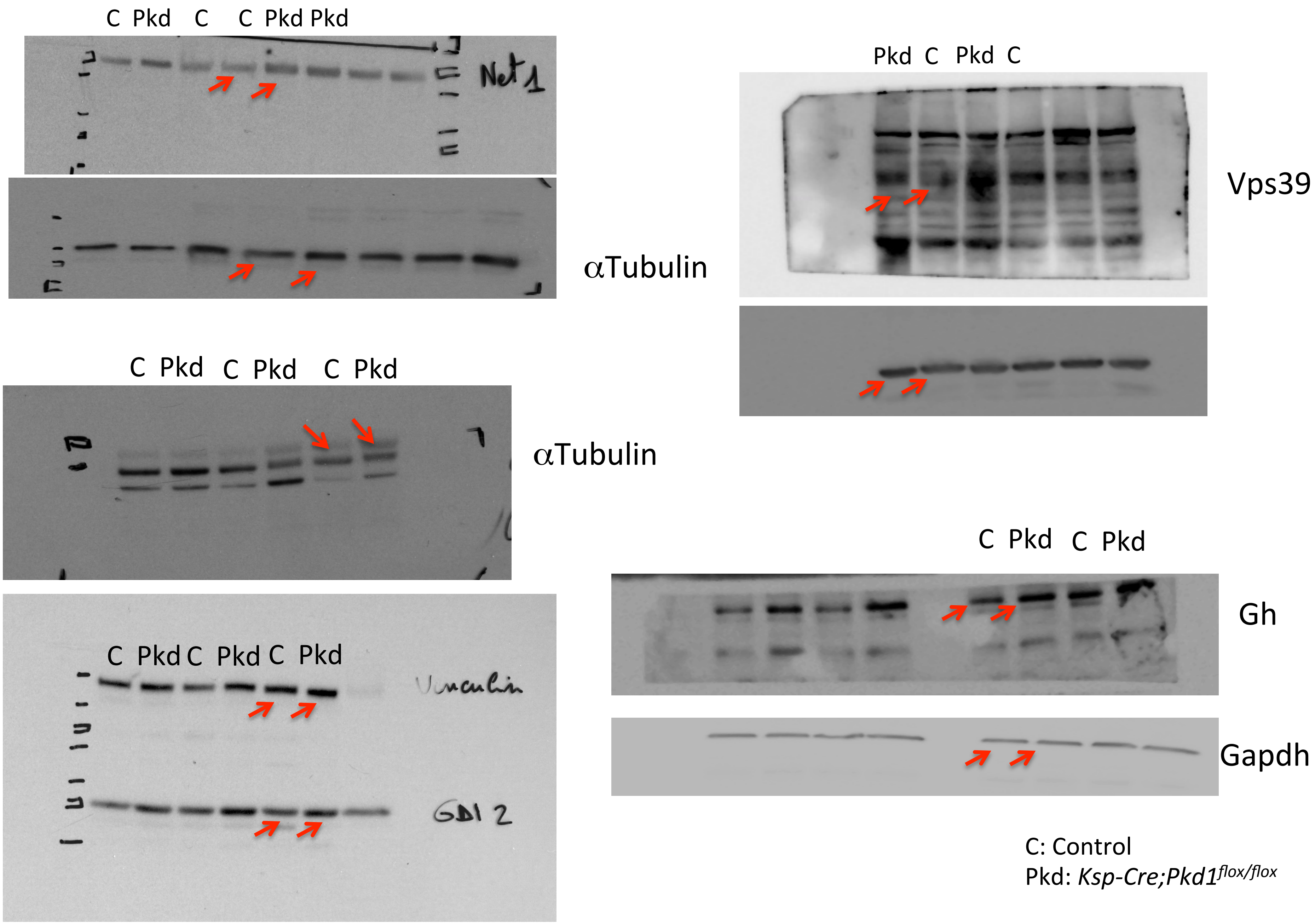

C: Control

Pkd: *Ksp-Cre;Pkd1*$^{flox/flox}$

Full unedited gel for figure 5A

The red arrows Indicate the bands reported in Figure 5A

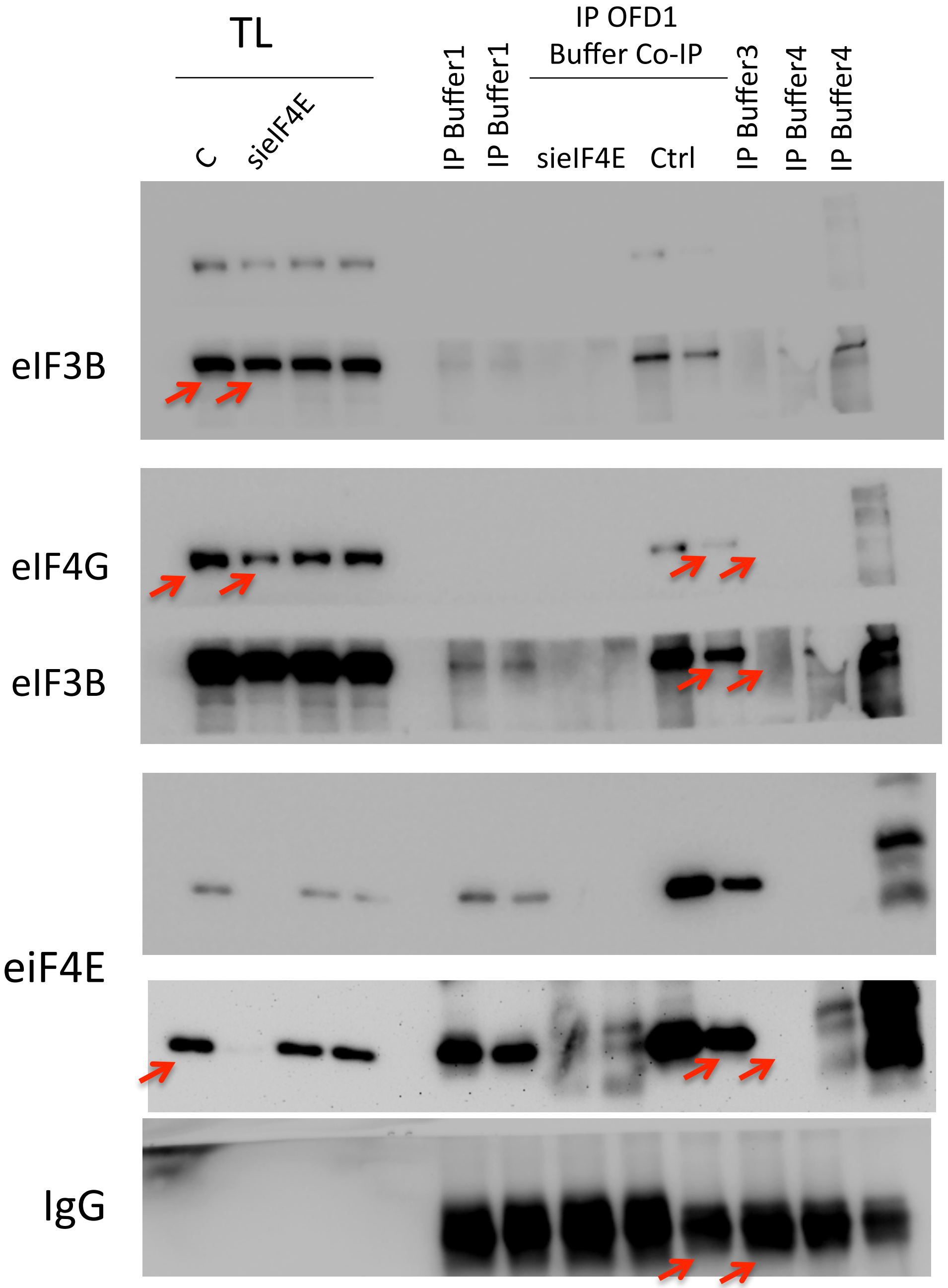

two different exposure of the same gel

two different exposure of the same gel

Full unedited gel for figure 5C

The red arrows Indicate the bands reported in Figure 5C

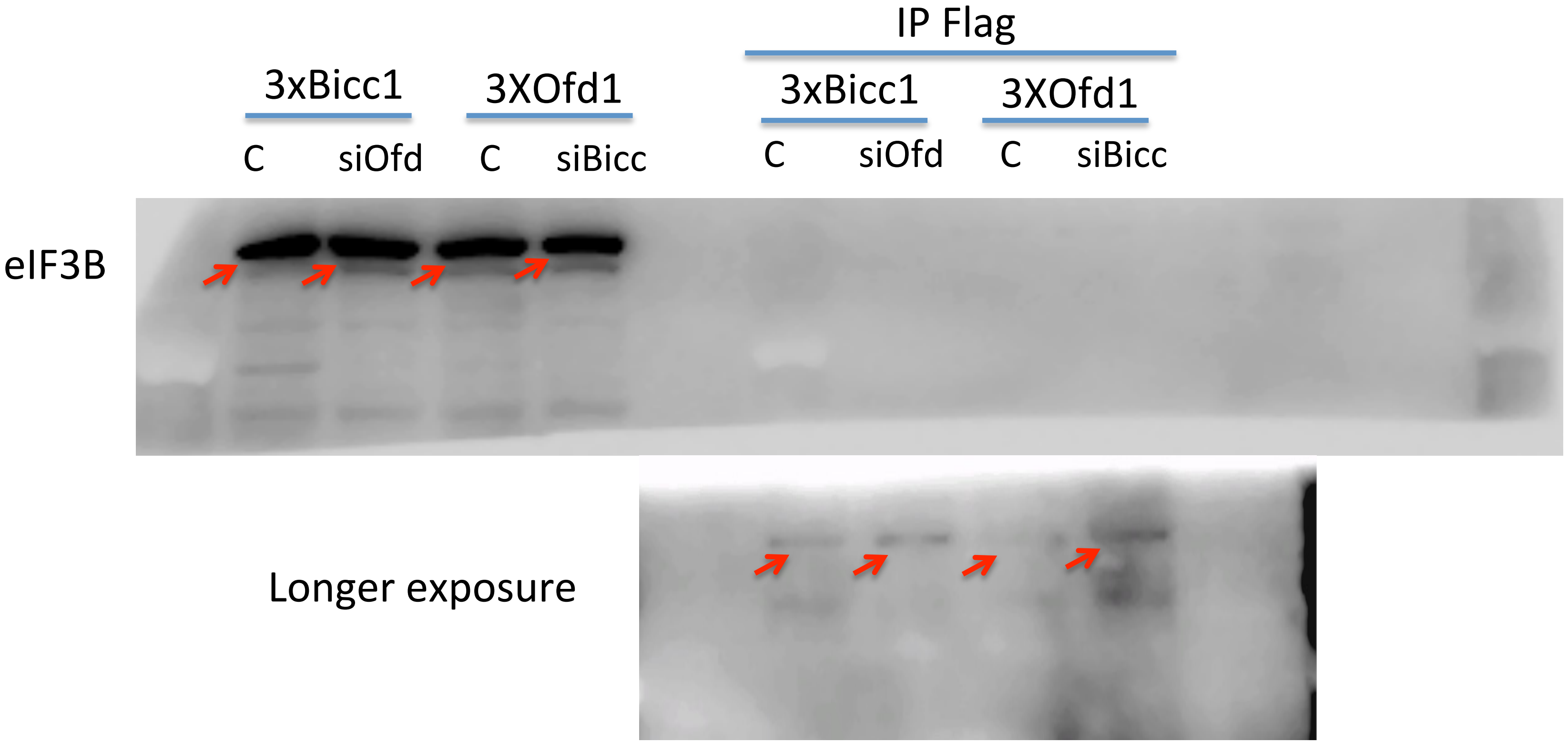

Supplement: Supplementary file 1 — Supplementary info [file 41598_2017_1156_MOESM1_ESM.pdf]
